# Proceedings of the CIMAGO Meeting – Challenges in Oncobiology Coimbra, 31st january 2020

**DOI:** 10.1097/MD.0000000000019821

**Published:** 2020-06-05

**Relevance of NF-kB and NRF2 transcription factors into multiple myeloma pathophysiology – A preliminary study**

Raquel Alves^1,2,3^, Ana Cristina Gonçalves^1,2,3^, Joana Jorge^1,2,3^, Catarina Afonso^4^, Adriana Roque^4^, Artur Paiva^2,5^, Letícia Ribeiro ^2,4^, Catarina Geraldes^1,2,4^, Ana Bela Sarmento-Ribeiro^1,2,3,4^

^*1*^*Laboratory of Oncobiology and Hematology and University Clinic of Hematologly, Faculty of Medicine of University of Coimbra, Portugal;*^*2*^*iCBR-Coimbra Institute for Clinical and Biomedical Research – Area of Environment, Genetics and Oncobiology (CIMAGO), Faculty of Medicine, University of Coimbra and CIBB - Center for Innovative Biomedicine and Biotechnology, University of Coimbra, Portugal;*^*3*^*CACC-Clinical Academic Center of Coimbra, Portugal;*^*4*^*Clinical Hematology Department, Centro Hospitalar e Universitário de Coimbra, Portugal;*^*5*^*Flow Cytometry Operational Management Unit, Clinical Pathology Service, Centro Hospitalar e Universitário de Coimbra, Portugal.*

Multiple myeloma (MM) is a mature B cell dyscrasia characterized by an abnormal proliferation of monoclonal plasma cells, the presence of CRAB symptoms and/or myeloma-defining events. Bone marrow's microenvironment and nuclear factor-κB (NF-kB) signaling pathway play a relevant role in MM pathogenesis, constituting now therapeutic targets. Furthermore, oxidative stress has been observed in several hematopoietic malignancies, being the NF-kB and the nuclear factor (erythroid-derived 2)-like 2 (NRF2) the two key transcription factors (TFs) that regulate the cellular responses to oxidative stress and inflammation. This work aims to elucidate the importance of NF-kB and NRF2 axis in the MM biology and response to treatment.

Thirty patients with MM were enrolled in the study and the bone marrow cell populations were evaluated by flow cytometry. The expression levels of NF-KB and its phosphorylated form, and of NRF2 were determined in the different cell populations, with a special focus on plasm cells, using flow cytometry. The TFs expression levels were correlated with clinical and laboratorial data.

Our cohort of 30 MM patients included 12 females and 18 males, with a median age at diagnosis of 62 years (32–74). Two patients at diagnosis, fourteen patients in remission and fourteen in relapse. According with international scoring system (ISS) 12 patients (40%) were in stage I, 10 patients (33.3%) in stage II, 5 patients (16.7%) in stage III and the ISS was not determined in 3 patients (10%). In tumor plasma cells, we observed at diagnosis a higher percentage of cells expressing NRF2 (22.9 ± 22.7) and NF-kB (21.7 ± 21.5) comparing with observed at remission time-point (NRF2: 7.0 ± 3.1; NF-kB: 4.3 ± 1.5). An intermediated expression was observed (NRF2: 12.2 ± 4.3; NF-kB: 6.5 ± 1.8) in samples at relapse. The increase of tumor plasma cells expressing these TFs was also associated with the presence of bone lesion and kappa light-chains. All the other bone marrow populations expressed these TFs, but higher expression was observed in granulocytes.

Our preliminary data suggest an association of NRF-2 and NF-kB expression with MM pathophysiology and disease stage.

This work was supported by CIMAGO and NRC-LPCC/CIMAGO grant.

**STAT5 – A new therapeutic target in acute lymphoblastic leukemia?**

Joana Jorge^1,2,3^, Alexandre Estevam^1^, Raquel Alves^1,2,3^, Beatriz Lapa^1,2,3^, José Manuel Nascimento-Costa^2,3,4^, Ana Cristina Gonçalves^1,2,3^, Ana Bela Sarmento-Ribeiro^1,2,3,5^

^*1*^*Laboratory of Oncobiology and Hematology and University Clinic of Hematology, Faculty of Medicine of University of Coimbra (FMUC), Coimbra, Portugal;*^*2*^*iCBR-Coimbra Institute for Clinical and Biomedical Research – Area of Environment, Genetics and Oncobiology (CIMAGO), Faculty of Medicine, University of Coimbra, CIBB - Center for Innovative Biomedicine and Biotechnology, University of Coimbra, Portugal;*^*3*^*CACC-Clinical Academic Center of Coimbra, Portugal;*^*4*^*University Clinic of Oncology/Faculty of Medicine of University of Coimbra (FMUC), Coimbra, Portugal;*^*5*^*Clinical Hematology Department/Centro Hospital e Universitário de Coimbra, Coimbra, Portugal*

The signal transducer and activator of transcription (STAT) family members regulate the transcription of multiple genes involved in cell survival, proliferation, differentiation and apoptosis. Deregulation of STATs was associated with several hematological neoplasias such as acute lymphoblastic leukemia (ALL). ALL is a malignant transformation and proliferation of lymphoid progenitor cells in the bone marrow, blood and extramedullary sites. Several genetic and epigenetic alterations are involved in ALL pathogenesis that induce a cellular homeostasis unbalance resulting in differentiation arrest, high proliferation rates and/or resistance to apoptosis. Pimozide (PIM) is a STAT5 inhibitor that presented an anti-neoplastic effect in several other tumors. With this study, we assessed the therapeutic potential of PIM in *in vitro* models of ALL.

To this end, two B-ALL cell lines (697 and REH) and two T-ALL (CEM and Jurkat) were incubated with PIM in single and daily administration schemes. Metabolic activity was evaluated by resazurin assay. Cell death was assessed by optic microscopy (May-Grünwald-Giemsa staining) and flow cytometry (FC), using the Annexin V and 7-AAD double staining. Cell cycle was evaluated by FC (IP/RNase). The expression levels of JAK/STAT target genes (*CCNB1*, *CCNE1*, *CCND1*, *BCL2*, *KI-67*, e *HPRT1)* were measured by qPCR. The statistical analysis was performed, considering a significance level of 95%.

Our results showed that PIM reduced the metabolic activity in a time, dose and cell line dependent manner. T-ALL cell lines were more sensitive to PIM, with an IC50, at 72 h, of 7.5 μM (both CEM and Jurkat) comparing to the observed in B-ALL cells (10 μM for both, 697 and REH). Daily administration of 2.5 μM PIM demonstrated to be more effective than single administration of 7.5 μM. PIM induced cell death mainly by apoptosis, as observed by FC and by morphological analysis, and also cell cycle arrest in G_0_/G_1_ phase in all cell lines. Incubation with PIM also reduced the expression of JAK/STAT targeted genes.

Our results suggest that PIM presents a therapeutic potential in *in vitro* ALL models and might be explored in other pre-clinical models.

This work was supported by FMUC, CIMAGO and FCT (SFRH/BD/51994/2012).

**Aldehyde dehydrogenase polymorphisms: The role in myelodysplastic syndromes and acute myeloid leukemia**

Duarte Silva^1^, Ana Cristina Gonçalves^1,2,3^, Bárbara Marques^1,4^, Joana Jorge^1,2,3^, Raquel Alves^1,2,3^, André Barbosa Ribeiro^2,4^,Emília Cortesão^1,2,3,4^, José M. Nascimento Costa^2,3,5^, Ana Bela Sarmento-Ribeiro^1,2,3,4^

^*1*^*Laboratory of Oncobiology and Hematology and University Clinic of Hematology, Faculty of Medicine, University of Coimbra,Portugal;*^*2*^*iCBR-Coimbra Institute for Clinical and Biomedical Research – Area of Environment, Genetics and Oncobiology (CIMAGO), Faculty of Medicine, University of Coimbra and CIBB - Center for Innovative Biomedicine and Biotechnology, University of Coimbra, Portugal;*^*3*^*CACC-Clinical Academic Center of Coimbra, Portugal;*^*4*^*Clinical Hematology Department/Centro Hospitalar e Universitário de Coimbra, Coimbra, Portugal;*^*5*^*University Clinic of Oncology/Faculty of Medicine, University of Coimbra, Coimbra, Portugal*

Aldehyde dehydrogenase (ALDH) is highly expressed in hematopoietic stem cells (HSC) and inhibition of ALDH promotes HSC self-renewal via reduction of retinoic acid activity. Since myelodysplastic syndrome (MDS) and acute myeloid leukemia (AML) pathogenesis is associated with genetic and epigenetic changes in HSC, dysregulation of processes related with differentiation and cellular proliferation of these stem cells may be associated with the development of these hematological neoplasias. Thus, this study aims to analyze the role of *ALDH1A2*, *ALDH3A1*, and *ALDH16A1* genetic variability in MDS and AML development and progression, in order to identify new potential risk factors and/or prognostic markers.

ALDH single nucleotide polymorphisms (SNP) (*ALDH1A2*: rs4646626, *ALDH3A1*: rs2228100, *ALDH3A1*: rs887241 and *ALDH16A1*: rs1320303) were genotyped using tetra-primer-ARMS-PCR, in 99 MN patients (49 MDS patients and 50 AML patients), and in 118 control individuals. The role of SNPs in MDS and AML susceptibility and development/progression was assessed by logistic regression analysis. The overall survival was analyzed by Kaplan-Meier method.

Our results show that *ALDH3A1* (rs2228100) CG genotype was associated with MN and MDS development (MN: OR=2.072, 95%CI 1.197–3.585, *p* = 0.009; MDS: OR = 2.204, 95%CI 1.119–4.344, *p* = 0.022), while GG carriers have 3.4x lower risk of MN development (OR = 0.296, 95%CI 0.105–0.828, p = 0.02). Additionally, *ALDH3A1* CG and GT haplotypes were associated with MDS risk (CG: OR = 1.901, 95%CI 1.181–3.062, p = 0.0104; GT: OR = 2.855, 95%CI 1.253–6.504, p = 0.0200), while CT haplotype carriers have 2.25x lower risk of MDS development (OR = 0.444, 95%CI 0.243–0.812, p = 0.0092). *ALDH1A2* (rs4646626) GG genotype were associated with higher risk of MDS progression. In AML group, the *ALDH1A2* (rs4646626) heterozygous carriers (AG) showed higher overall survival than homozygous carriers (AA + GG) (HR = 0.513, 95%CI 0.273–0.966, p = 0.035).

Altogether, these findings reinforce the association of ALDH with MN susceptibility. The participation of ALDHs in oxidative stress and mutagenesis, contribute to the role of these enzymes in MN development, progression and prognosis.

***IREB2* rs17483548 single nucleotide polymorphism is associated with colorectal cancer**

Rui Gomes^1^, Ana Cristina Gonçalves^1,2,3^, Joana Jorge^1,2,3^, Raquel Alves^1,2,3^, Amélia Pereira^1,4^, Ana Bela Sarmento-Ribeiro^1,2,3,5^, José M. Nascimento Costa^2,3,6^

^*1*^*Laboratory of Oncobiology and Hematology and University Clinic of Hematology/Faculty of Medicine, University of Coimbra, Portugal;*^*2*^*iCBR-Coimbra Institute for Clinical and Biomedical Research – Area of Environment, Genetics and Oncobiology (CIMAGO), Faculty of Medicine, University of Coimbra and CIBB - Center for Innovative Biomedicine and Biotechnology, University of Coimbra, Portugal;*^*3*^*CACC-Clinical Academic Center of Coimbra, Portugal;*^*4*^*Medicine Department, Hospital Distrital da Figueira da Foz, EPE, Figueira da Foz, Portugal;*^*5*^*Clinical Hematology Department/Centro Hospitalar e Universitário de Coimbra, Coimbra, Portugal;*^*6*^*University Clinic of Oncology/Faculty of Medicine, University of Coimbra, Coimbra, Portugal*

Colorectal cancer (CRC) is one of the most common cancer worldwide. The CRC risk is determined by a complex interaction between environmental exposures and genetic variants. A growing body of evidence has proved that iron overload and dietary iron are associated with CRC. Considering the fundamental role of iron-regulatory protein 2 (IRP2) in the regulation of intracellular iron homeostasis, it is reasonable that some polymorphisms in *IREB2* gene, which encode IRP2, could be involved in CRC carcinogenesis. In this context, we investigated the role of *IREB2* rs17483548 single nucleotide polymorphism in CRC susceptibility, location, staging, and prognosis, in order to identify new potential risk factors and/or prognostic markers.

To this end, a hospital-based case-control study with 83 CRC patients and 176 healthy controls was conducted. DNA from patients and controls was extracted from whole blood samples and the *IREB2* rs17483548 corresponding genomic region was amplified by tetra-primer ARMS-PCR assay. The role of this variant in CRC susceptibility was performed by logistic regression analysis. The overall survival of patients stratified according to their genotypes was analyzed by Kaplan-Meier method (log-rank test and Cox proportional hazards model).

The results showed that GA genotype (OR = 0.522, 95% CI 0.287–0.951, *p* = 0.034) and AA genotype (OR = 0.361, 95% CI 0.167–0.782, *p* = 0.010) were associated with a decreased susceptibility for CRC. On the other hand, GG genotype (OR = 2.133, 95% CI 1.212–3.755, *p* = 0.009) was found to increase the risk of CRC development. Additionally, GA and AA genotypes were associated with an increased predisposition for locoregional CRC. In contrast, GG genotype decreases the risk of right and transverse colon neoplasms. However, CRC patient's overall survival was not influenced by this genetic variant (HR = 1.269; 95% CI 0.615–2.620; *p* = 0.519).

Altogether, these results suggest that *IREB2* (rs17483548) single nucleotide polymorphism is associated with CRC development and may play an important role in tumor susceptibility and staging. However, more studies are needed to better characterize the impact of this single nucleotide polymorphism in CRC.

**NRF2 modulation as a new therapeutic approach in AML – In *vitro* studies**

Diana Figueiredo^1^, Joana Jorge^1,2,3^, Raquel Alves^1,2,3^, Beatriz Lapa^1,2,3^, Ana Cristina Gonçalves^1,2,3^, Ana Bela Sarmento-Ribeiro^1,2,3,4^

^*1*^*Laboratory of Oncobiology and Hematology and University Clinic of Hematology, Faculty of Medicine, University of Coimbra, Portugal;*^*2*^*iCBR-Coimbra Institute for Clinical and Biomedical Research – Area of Environment, Genetics and Oncobiology (CIMAGO), Faculty of Medicine, University of Coimbra and CIBB - Center for Innovative Biomedicine and Biotechnology, University of Coimbra, Portugal;*^*3*^*CACC-Clinical Academic Center of Coimbra, Portugal;*^*4*^*Clinical Hematology Department, Centro Hospitalar e Universitário de Coimbra, Portugal*

Oxidative stress (OS), characterized by an imbalance between the production of reactive oxygen species (ROS) and the biological system's ability to neutralize them, has been observed in most cancers, including acute myeloblastic leukemias (AML). An important mechanism in cellular defense against OS is the NRF2 pathway. This transcription factor protects normal cells from malignant transformation, regulating the expression of several antioxidant and detoxification genes. However, once malignant transformation occurs, NRF2 protects tumor cells from OS and preventing chemotherapy-induced cytotoxicity. Understanding the dual role of NRF2 is essential to consider it as a potential therapeutic target in AML. The aim of the present study was to analyze the effect of NRF2 activation and inhibition on AML cell lines.

To achieve these goals, two AML cell lines (HEL and THP-1) were culture in absence and presence of ML385 (NRF2 inhibitor) and Oltipraz (NRF2 activator). The metabolic activity was evaluated by resazurin assay and cell death by optical microscopy (May-Grunwald staining) and flow cytometry (FC; Annexin V/7-AAD double staining). Cell cycle analysis was evaluated by FC, using a PI/RNAse solution. The intracellular levels of ROS and GSH were quantified by FC (DCFH_2_-DA, DHE and MO probes). The expression of NRF2-regulated genes (*NFE2L2*, *KEAP1*, *NQO1*, *NFKB1*) was tested by qPCR. The results were statistically analyzed considering a level of significance of 95% (p < 0.05).

The results showed that both Oltipraz and ML385 decreased the metabolic activity of all cells, with ML385 showing a higher effect compared to Oltipraz (ML385 IC_50_ 72 h: HEL = 30 μM, THP-1 = 65 μM; Oltipraz IC_50_ not reached). Both drugs induced a cytotoxic effect mediated by apoptosis. In THP-1 cells, a G_0_/G_1_ arrest were also observed. Additionally, cells incubated with both drugs showed an increase in the ROS/GSH ratio and in *KEAP1* and *NQO1* expression levels suggesting that Oltipraz may not be a NRF2 activator, at least in AML cells.

The results suggest that AML cells are sensitive to NRF2 modulation and Oltipraz and ML385 may represent new potential therapeutic approaches in these hematological neoplasms.

**Epigenetics in chronic lymphocytic leukemia - Role in pathogenesis and treatment**

José Pedro Carda^1,2,3,4^, Raquel Alves^1,2,3^, Joana Jorge^1,2,3^, Sara Duarte^4^,Amélia Pereira^2,5^, Ilda P. Ribeiro^2,3,6^, Letícia Ribeiro^2,4^, Ana Cristina Gonçalves^1,2^, Isabel Marques Carreira^2,5^, Ana Bela Sarmento-Ribeiro^1,2,3,4^

^*1*^*Laboratory of Oncobiology and Hematology and University Clinic of Hematology, Faculty of Medicine, University of Coimbra, Portugal;*^*2*^* iCBR-Coimbra Institute for Clinical and Biomedical Research – Area of Environment, Genetics and Oncobiology (CIMAGO), Faculty of Medicine, University of Coimbra and CIBB - Center for Innovative Biomedicine and Biotechnology, University of Coimbra, Portugal;*^*3*^*CACC-Clinical Academic Center of Coimbra, Portugal;*^*4*^*Clinical Hematology Department, Centro Hospitalar e Universitário de Coimbra, Portugal;*^*5*^*Medicine Department, Hospital Distrital da Figueira da Foz, Figueira da Foz, Portugal;*^*5*^*Laboratory of Cytogenetics and Genomics /Faculty of Medicine, University of Coimbra, Coimbra, Portugal*

Chronic lymphocytic leukemia (CLL) is the most common leukemia in the Western World. In recent years, more interest has been focused on the contribution of epigenetic changes to CLL development. In this context, the main goal of this work was to clarify the involvement of epigenetic modifications in CLL development, and correlate them with patients’ clinical features, survival and prognostic risk groups as well as to analyze the therapeutic potential of epigenetic modulators.

The methylation status of 25 tumor suppressor genes (ME001B, MRC-Holland) was carried out in 24 CLL patients with a median age of 70 years (range 50–81), 25% (n = 6) females and 75% (n = 18) males. Regarding Binet staging system, 19 (79%) were low risk (A and B) and five (21%) were high risk (C). Peripheral blood mononuclear cells (PBMCs) obtained from CLL patients were incubated in the absence and presence of decitabine (DAC), azacitidine (5-AC), vorinostat (SAHA) and/or panobinostat (PAN) in several concentrations, during 72 h. Cell viability will be evaluated by fluorometric microculture cytotoxicity assay (FMCA) every 24 h. Cell death were analyzed by flow cytometry (annexin V/7-AAD double stain).

The results show that CLL patients have methylation in 13 of the 25 tumor suppressor genes studied, whereas controls only show methylation in the *MSH6* gene [6/13 (46%)]. CLL patients have eight of these genes significantly hypermethylated [*PAX5*: 9/24 (38%); *KLLN*: 12/24 (50%); *WT1*: 22/24 (92%); *CADM1*: 9/24 (38%); *THBS1*: 13/24 (54%); *CDH13*: 15/24 (54%); *TP53*: 9/24 (38%); *GATA5*: 16/24 (67%)]. In addition, 58% (14/24) of CLL patients have 5 or more methylated genes and high-risk patients have a higher number of methylated genes (11/25) than low-risk patients (8/25). Epigenetic modulation revealed promising results in monotherapy. All drugs reduced the viability of CLL PBMCs (IC_50_: 30–40 μM 5-AC, 40–50 μM DAC, 50 nM PAN, 2.5–5 μM SAHA), inducing cell death by apoptosis with high specificity for neoplastic B cells. PAN and SAHA also induced a cytostatic effect.

This study suggests that DNA methylation is a common event in CLL patients and that epigenetic modulators induce neoplastic B cells death.

**Therapeutic effect of Oltipraz in acute lymphoblastic leukemia – Preliminary *in vitro* studies**

Nisa Magalhães^1,2^, Diogo Roque^1,2,^; Maria Inês Costa^2,^ Eduardo Gomes^2,^ Joana Jorge^2,3,4^, Raquel Alves^2,3,4^, Beatriz Lapa^2,3,4^, Ana Cristina Gonçalves^2,3,4^, Ana Bela Sarmento-Ribeiro^2,3,4,5^

^*1*^*Department of Chemistry, Faculty of Science and Technology, University of Coimbra;*^*2*^*Laboratory of Oncobiology and Hematology and University Clinic of Hematology, Faculty of Medicine, University of Coimbra, Portugal;*^*3*^*CACC-Clinical Academic Center of Coimbra, Portugal;*^*4*^* iCBR-Coimbra Institute for Clinical and Biomedical Research – Area of Environment, Genetics and Oncobiology (CIMAGO), Faculty of Medicine, University of Coimbra and CIBB - Center for Innovative Biomedicine and Biotechnology, University of Coimbra, Portugal;*^*5*^*Clinical Hematology Department, Centro Hospitalar e Universitário de Coimbra, Portugal*

Acute lymphoblastic leukemia (ALL) is the most common hematological neoplasia in childhood, characterized by excessive proliferation of immature lymphoid cells and differentiation arrest, resulting in the accumulation of lymphoblasts and, consequently, the in suppression of normal hematopoiesis. In ALL there is an increase of reactive oxygen species (ROS) production and a decrease in antioxidant defenses, which makes evident the involvement of oxidative stress in the disease. The KEAP1-NRF2 pathway is an important mediator of the cytoprotective response to exogenous and endogenous stress induced by ROS. One of the major signaling proteins within this pathway is the transcription factor NRF2, which plays a vital role in cellular defense against oxidative stress through activation of various antioxidant genes. Thus, the aim of this study is to investigate the therapeutic potential of Oltipraz, an activator of NRF2, in T- and B-ALL cell lines.

In this context, T-ALL (MOLT-4 and CEM) and B-ALL cell lines (697 and KOPN8) were cultured in the absence and presence of increasing concentrations of Oltipraz in monotherapy for 72 h. Metabolic activity was determined by the resazurin assay. Cell death was assessed with flow cytometry by double labeling with Annexin V and 7-AAD and through morphological analysis by Giemsa staining. Cell cycle was evaluated by flow cytometry using PI/RNase solution. The results were statistically analyzed considering a significance level of 95% (p < 0.05).

Preliminary results showed that Oltipraz reduced metabolic activity in a time-, dose- and cell line dependent manner. After 72 h of treatment, B-ALL cells were more sensitive to Oltipraz (697: IC_50_ = 27.5 μM, KOPN8: IC_50_ = 4.5 μM) compared to T-ALL cells (MOLT-4: IC_50_ = 87.3 μM, CEM: IC_50_ = 40.1 μM). Oltipraz induced cell death by apoptosis in both B- and T-ALL subtypes (697 and MOLT-4).

In summary, these preliminary results suggest that Oltipraz has an antiproliferative effect and that ALL-B cells are more sensitive to this drug. In addition, Oltipraz induces some cell death by apoptosis in LLA-T (MOLT-4) and LLA-B (697) cells.

**Zinc as a genoprotective player of DNA damage response – Preliminary results**

Maria Inês Costa^1^, Nisa Magalhães^1,2^; Diogo Roque^1,2^; Eduardo Gomes^1^, Raquel Alves^1,3,4^, Joana Jorge^1,3,4^, Beatriz Lapa^1,3,4^, Mafalda Laranjo^3,4,5^, Maria Filomena Botelho^3,4,6^, Ana Bela Sarmento-Ribeiro^1,3,4,5^, Ana Cristina Gonçalves^1,3,4^

^*1*^*Laboratory of Oncobiology and Hematology and University Clinic of Hematology, Faculty of Medicine, University of Coimbra, Portugal;*^*2*^*Department of Chemistry, Faculty of Science and Technology, University of Coimbra;*^*3*^* iCBR-Coimbra Institute for Clinical and Biomedical Research – Area of Environment, Genetics and Oncobiology (CIMAGO), Faculty of Medicine, University of Coimbra and CIBB - Center for Innovative Biomedicine and Biotechnology, University of Coimbra, Portugal;*^*4*^*CACC-Clinical Academic Center of Coimbra, Portugal;*^*5*^*Clinical Hematology Department, Centro Hospitalar e Universitário de Coimbra, Portugal;*^*6*^*Biophysics Institute/Faculty of Medicine, University of Coimbra, Portugal*

Micronutrients are important regulators of genomic stability, intervening in relevant pathways of the DNA damage response (DDR) as substrates and cofactors of numerous enzymes. Micronutrients’ deficiency was already shown to cause similar effects as those produced by well-known genotoxic agents. Therefore, the adequate provision of micronutrients may be determinant for efficient DNA repair. Zinc is a vital micronutrient and a component of more than 300 enzymes involved in DDR. Increasing evidence has pointed its importance in DNA integrity and repair. The main aim of this study was to investigate the effects of zinc supplementation in cellular ability to handle genotoxic stimuli and DNA repair.

The experiments were conducted on one human acute myeloid leukemia cell line (HEL) and on normal human lymphocytes (IMC), incubated for 2 days in RPMI-1640 medium with FBS (non-supplemented standard medium), zinc-depleted FBS and FBS plus 40 μM ZnSO_4_. Cells were then exposed to 10 μM H_2_O_2_ for 30 minutes. Chromosomal damage was analyzed using the cytokinesis-block micronucleus cytome assay. Two hours after H_2_O_2_ treatment, DNA repair was accessed with the alkaline comet assay.

Our preliminary data shown that zinc supplementation resulted in a lower frequency of micronuclei and other chromosomal abnormalities in HEL and IMC (10 ± 1% and 7.8 ± 0.8%, respectively) when compared to non-supplemented conditions (14 ± 0% and 11.8 ± 0.3%). Zinc-depleted cells presented the highest frequency of chromosomal damage (20 ± 1% and 16.3 ± 3%), suggesting that zinc deficiency might affect cellular ability to cope with genotoxic stimuli. Nuclear buds were the most frequently found lesions in non-supplemented and zinc supplemented cells, whereas zinc depletion resulted mainly in micronuclei. Regarding the analysis of DNA repair, an initial comet assay on IMC shown a reduced DNA repair ability in cells cultured in zinc depletion and a slight recovery in cells from non-supplemented and supplemented mediums.

These preliminary results suggest a protective role for zinc on chromosomal damage and a defective DDR when zinc is absent.

**EPO-EPOR axis as a prognostic and drug response biomarker in myelodysplastic syndrome – Preliminary results**

Beatriz Lapa^1,2^, Raquel S. Alves^1,2,3^, Joana Jorge^1,2,3^, Emília Cortesão^1,2,3,4^, Amélia Pereira^2,5^, Letícia Ribeiro^2,3,4^, Ana Cristina Gonçalves^1,2,3^, Ana B. Sarmento-Ribeiro^1,2,3,4^

^*1*^*Laboratory of Oncobiology and Hematology and University Clinic of Hematology, Faculty of Medicine, University of Coimbra, Portugal;*^*2*^* iCBR-Coimbra Institute for Clinical and Biomedical Research – Area of Environment, Genetics and Oncobiology (CIMAGO), Faculty of Medicine, University of Coimbra and CIBB - Center for Innovative Biomedicine and Biotechnology, University of Coimbra, Portugal;*^*3*^*CACC-Clinical Academic Center of Coimbra, Portugal;*^*4*^*Clinical Hematology Department, Centro Hospitalar Universitário de Coimbra;*^*5*^*Medicine Department, Hospital Distrital da Figueira da Foz, Portugal*

Myelodysplastic syndromes (MDS) are a heterogeneous group of clonal hematopoietic stem cell disorders characterized by peripheral blood cytopenias, ineffective hematopoiesis, dysplasia and tendency to acute myeloid leukemia transformation. Anemia is the more frequent cytopenia that could be treated erythropoietin (EPO) stimulating agents (ESA). EPO is the primary regulator of erythropoiesis and has pleiotropic effects. EPO analogs induce erythroid responses in few low-risk MDS patients and some develop neutralizing anti-EPO or anti-EPO receptor (EPOR) antibodies. The aim of this work was to asses EPO and EPOR expression in MDS patients in order to identify novel prognostic and drug response biomarkers.

Six peripheral blood samples were collected from MDS patients, and stratified according to recombinant EPO treatment response, patients that respond to EPO treatment (n = 2) and patients that do not respond (n = 4). Flow cytometry was used to identify HSC and progenitor cells and assess the expression of EPO and EPOR. RNA from the samples was isolated and used to evaluate the expression levels of *EPO* and *EPOR* by qPCR.

The non-responder patients presented a lower percentage of CD34+/CD117+ cells expressing EPOR (4.3 ± 1.4) as well as lower expression levels of EPOR (1810 ± 537) when compared do EPO responders (13.4 ± 1.8 and 4957 ± 414, respectively). The more differentiated progenitor cells (CD34+/CD45dim/CD117+/HLA-/CD13+/CD123+/CD105-) from non-responder patients presented a higher percentage of cells expressing EPOR (23.7 ± 4.8%) compared to responders (2.3 ± 0.5%) and expression levels like EPO responders (responders: 708 ± 65 MIF; non-responders: 772 ± 81 MIF). Furthermore, the responder patients presented an *EPO* gene expression 4-fold higher (0.50 ± 0.08) than the non-responders (0.13 ± 0.02) but *EPOR* gene expression presented a similar value (responders: 3.9 ± 0.3; non-responders: 4.3 ± 1.1).

EPO responders and non-responders’ MDS patients appeared to have different percentage of cells that express EPOR as well as different expression levels of *EPO.* Therefore, these preliminary results suggest that EPO/EPOR axis could be used as a predictive biomarker of therapy response.

This project was funded by Liga Portuguesa Contra o Cancro and Sociedade Portugusa de Hematologia.

**The clinical value of Next-Generation Sequencing in Acute Myeloid Leukemia**

Bárbara Marques^1,2^, Adriana Roque^1,2,3^, Carolina Afonso^1^, Daniela Coelho^1^, João Gomes^1^, Ana Teresa Simões^1^, Margarida Coucelo^1,2,3,4^, Sandra Marini^1^, Letícia Ribeiro^1,3,4^, Ana Isabel Espadana^1^, Emília Cortesão^1,2,3,4^

^*1*^*Clinical Hematology Department, Centro Hospitalar e Universitário de Coimbra, Portugal;*^*2*^*Faculty of Medicine of University of Coimbra, Portugal;*^*3*^*iCBR-Coimbra Institute for Clinical and Biomedical Research – Area of Environment, Genetics and Oncobiology (CIMAGO), Faculty of Medicine, University of Coimbra and CIBB - Center for Innovative Biomedicine and Biotechnology, University of Coimbra, Portugal;*^*4*^*CACC-Clinical Academic Center of Coimbra, Portugal;*

**Contex:** Next-Generation Sequencing (NGS), which analyze several genes at once, is useful in diseases with recurrent mutations. As Acute Myeloid Leukemia (AML) is highly heterogeneous, genetic profile study is worthwhile.

**Aim:** to evaluate mutation pattern in AML with cytogenetic normal and not available karyotype.

**Methods:** Characterization of mutation profiling by NGS in AML patients and correlation with outcome. **Results:** We analyzed 28 patients, 18 males, with median age 60(52–69) years. Karyotype was normal in 19 and not available in 9 patients. At least 1 driver mutation per patient was detected, median of 4 mutations (1–8), and 18 patients (64.3%) with ≥3 mutations. It was found mutations in *NPM1*[16], DNA methylation (*DNMT3A*[12], *TET2*[7], *IDH1*[4], *IDH2*[4]), cohesin complex (*KMT2A*[2], *BCOR*[4], *STAG2*[2], EZH2[1], *SMC3*[1]), splicing (*SRSF2*[1], *SF3B1*[1]), signaling pathway (*NRAS*[7], *FLT3*[10], *KIT*[1], *PTPN11*[5], *CBL*[1], *JAK2*[1]) and transcription factors (*RUNX1*[5], *GATA1*[1], *GATA2*[1], *CUX1*[1], *ETV6*[1]). *NPM1* mutation was associated more frequently to *DNMT3* mutation (Odds Ratio = 8.3, 95%confidence interval [CI] = 1.3–51.7, P = 0.015). According to WHO2016, there were 17 AML with mutated *NPM1* (m*NPM1*), 7 AML not otherwise specified (NOS), 2 AML with myelodysplasia-related changes (MRC) and 2 AML with mutated *RUNX1* (m*RUNX1*) patients. Based on genomic status, 13(46.4%) patients were reclassified. All NOS were reclassified to AML with mutated chromatin, RNA-splicing genes, or both (Chr/Spl)[2] and AML with driver mutations but no detected class-defining lesions (Mut/NoClass)[5]. Two m*NPM1*, 2 m*RUNX1* and 2 MRC patients were also reclassified into Chr/Spl[6]. From 22(78.6%) patients treated with intensive chemotherapy, 59.1% reached complete remission. In multivariate analysis, ≥3 mutations were associated with higher mortality (Hazard Ratio[HR] = 17.8; 95%CI = 2–158; P = 0.010), as well as Mut/NoClass (HR = 58.1; 95%CI = 3–1123; P = 0.007).

**Conclusions:** NGS, a single and fast methodology, is an important clinical tool, allowing to reclassify AML with prognostic impact. Providing mutation pattern, NGS could identify patients with potential benefit from new molecular targeted therapy.

**Prescription pattern in palliative and end of life care**

Tatiana Peralta^1^, Isabel Vitória Pereira^2^, Paulo Reis Pina^3,4^, Marília Dourado^1,5^

^*1*^*Faculdade de Medicina. Universidade de Coimbra-Portugal;*^*2*^*Faculdade de Farmácia. Universidade de Coimbra-Portugal;*^*3*^*Unidade de Cuidados Paliativos Poverello - Domus Fraternitas, Braga Portugal;*^*4*^* Faculdade de Medicina. Universidade de Lisboa.-Portugal.*^*5*^*Centro de Estudos e Desenvolvimentos de Cuiddaos Continuados e Paliativos- Faculdade de Medicina. Universidade de Coimbra.Portugal*

**Context/aims**: Symptom control and quality of life are essential in palliative care (PC), mainly at the end of life. Therapeutic re-evaluation is a need under these circumstances. This study aims to assess the prescribing pattern in a PC unit in two different moments: admission and day of death.

**Methods**: A single observational retrospective centre study was carried out from August 2017 to December 2018 in a PC unit. Sociodemographic data, diagnoses, doses, frequency and administration route of drugs on admission and on the day of death were collected.

**Results:** Clinical records of 119 adult who died during the study period were analysed. The mean age was 70 (39–101) years old, 53.8% were male. The most common pathology, 84.0%, was cancer, of which 56.0% were at an advanced stage. At admission, 8.01 (2–22) Regular Drugs (RD) and 2.75 (0–8) *pro re nata* (PRN) drugs were prescribed on average. On the day of death there was a decrease prescribed drugs to 4.93 (0–13) and 3.90 (1–9), respectively. The most prescribed pharmacological class on admission and death, as RD or PRN, were opioids (77.3% and 84.8%; 75.6% and 92.4% respectively). Laxatives (74.8%) and anti-ulcer drugs (68.9%) are the second and the third most regularly prescribed classes at admission, while at death are antispasmodics (52.1%) and corticosteroids (44.5%). Morphine (86.4%, 91.6%), acetaminophen (43.7% and 47.9%), levomepromazine (1.7% and 43.7%) and midazolam (19.3% and 29.4%) were the most prescribed in PRN on admission and death. At death, there is a tendency to increase prescription of butylscopolamine, midazolam, diazepam, levomepromazine and dexamethasone. The most frequent route of administration of drugs were oral at admission and subcutaneous at death.

**Conclusions**: Polypharmacy is a real problem in palliative care. A reduction of prescriptions was verified from admission to death, mainly due to the reduction of the use of comorbid-based drugs. Further studies for the end of life prescription are required.

**Advance Care Planning: why does it matter? - a systematic review**

Adriana Santos^1^, Marília Dourado^2^

^*1*^*Mestre em Medicina. Faculdade de Medicina da Universidade de Coimbra, Portugal;*^*2*^*Centro de Estudos e Desenvolvimentos de Cuidados Continuados e Paliativos - Faculdade de Medicina da Universidade de Coimbra, Portugal.*

**Context/Aim:** Advance Care Planning (ACP) can be defined as a process that allows individuals, who have the ability to decide, to indicate and plan future healthcare in the event of decision disability. It is a communication process among patients, families and healthcare professionals, where prognostic information, therapeutic options, patient's life goals, values and wishes for further treatment are addressed, in order to respect patient preferences. Although interest in this topic has grown in recent years, the positive effects that this intervention may have are not evident yet. Therefore, the aim of this work is to identify the main benefits associated with ACP.

**Methods:** A search for articles on ACP published in English between January 2010 and September 2018 has been carried out in MEDLINE, Embase and Cochrane Library databases. Further articles relevant to this subject have been found when revising the reference list. The research resulted in 377 articles, of which 24 were included in this study after meeting inclusion criteria. Thirteen refer to experimental studies, 10 to observational studies, and 1 to cost-effectiveness study.

**Results:** Advanced care planning, by facilitating a discussion centred on the identification of individual's moral values and beliefs, allowed a more informed decision-making and a stable end-of-life treatment preference without increasing patient anxiety or depression. ACP-induced communication conduced to a greater understanding and compliance with the patient's treatment preferences and developed the confidence of family members and healthcare professionals in decision-making. ACP seems to have contributed to reduce the number of hospital deaths, particularly in intensive care units, fewer bereavement complications, to promote legal documentation and to reduce the costs associated with health care.

**Conclusion:** The multiple benefits associated with ACP reinforce the need to create a programme for a systematic implementation of this type of care.

**Family conference: a quality tool in the communication process in palliative care**

Ana M. N. Rocha^1^, Marília A. R. F. Dourado^2^, Manuel L. V. Capelas^3^

^*1*^*University of Lisbon, Portuguese Oncology Institute of Coimbra Francisco Gentil, EPE, Coimbra, Portugal.*^*2*^*Faculty of Medicine University of Coimbra, Portugal;*^*3*^*Health Science Institute of Catholic Portuguese University, Lisbon Portugal.*

**Introduction**: Family Conference (FC) is a quality indicator in palliative care (PC), based in communicational and rational process to managing the emotion and desires of patients, patient's family and health care team^1,2^. It is important to understand the effectiveness of FC in PC and what is the level of satisfaction achieved by each decision makers of the Advanced Care Plan: patient, family and health professionals’ team.

**AIM**: Evaluate the overall satisfaction of each participants in the FC and which variables influence this outcome.

**Material & methods**: We performed an exploratory, quantitative, observational, analytical and cross-sectional study. We made an accidental non-probabilistic sampling of 94 FCs in Private Palliative Care Unit, Palliative Care Unit of the National Palliative Care Network, Community Palliative Care Support Unit and Intrahospital Palliative Care Support Unit. To evaluate the levels of satisfaction, we use a questionnaire “Satisfaction Assessment with FC” with a Cronbach's Alpha > 0.75. The results were analyzed using the Mann-Whitney and Kruskall-Wallis tests. Sampling: 94 FCs, 93 health professionals’ as FC managers, 83 family members, 24 patients.

**Results:**. 98.2% of the CF objectives were assessed as satisfied or very satisfied. Statistically significance were detected in the overall satisfaction of the participants with the FC (H = 9.463; p = 0.009), with a higher patient and family satisfaction index than the professionals. The variables planning, PC typology, professional category of FC manager, place of care, relative's kinship and patient presence had a significant influence on the level of satisfaction of participants in FC. The number of professionals in the FC, the duration and the space where the FC was performed did not show any influence on satisfaction rate.

**Conclusion:** The FC has been shown to be effective in terms of the goals set in all PC services typologies and very satisfactory to the participants. Patient and family value having an opinion in the health process and appreciate listening to professionals ^3,4,5^. Therefore, Family conference should be use as a quality tool in the communication process in Palliative Care.

**Risk factors for morbidity after ALPPS procedure in the treatment of colorectal liver metastases (CRLV)**

Rodrigo Nemésio^1,2^, Ricardo Martins^1,2,3,4^, Henrique Alexandrino^1,2^, Marco Serôdio^1,2^, António Pinho^1,2^, Ana Velez^1,2^, Hamilton Batista^1^, José Guilherme Tralhão^1,2,3,4^

^*1*^*Serviço de Cirurgia Geral do Centro Hospitalar e Universitário de Coimbra;*^*2*^*Clínica Universitária de Cirurgia da Faculdade de Medicina da Universidade de Coimbra;*^*3*^* iCBR-Coimbra Institute for Clinical and Biomedical Research – Area of Environment, Genetics and Oncobiology (CIMAGO), Faculty of Medicine, University of Coimbra and CIBB - Center for Innovative Biomedicine and Biotechnology, University of Coimbra, Portugal;*^*4*^*CACC-Clinical Academic Center of Coimbra, Portugal;*

The ALPPS procedure allows for the treatment of bilobar CRLM in a short period of time, reducing drop-out rates and disease progression between stages. A better selection of patients and recent technical developments have been decisive in decreasing the morbidity and mortality rates that were initially associated with this procedure. We aim to analyze the results of our Center in the treatment of CRLM using the ALPPS technique and identify risk factors associated with higher morbidity.

Between 2015 and 2019, 18 patients (63,8 ± 8,5 years old; 4F:14 M) with a median of 10,5 CRLM underwent ALPPS. Morbidity was defined according to Clavien-Dindo Classification; survival was estimated using the Kaplan-Meier method; and a multivariate logistic regression model was used for the risk factor analysis using SPSS.

The mean survival was 23,12 ± 3,88 months (65,8%/1year and 28,7%/3 years). 8 patients (46,7%) experienced disease recurrence and the mean disease-free survival was 17,26 ± 2,38 months. Overall morbidity rates were 50% (9 patients) – 38,9% reported major complications (Clavien-Dindo≥3). As risk factors for major morbidity we identified the presence of more than 10 metastatic lesions, more than 10 cycles of chemotherapy before surgery and overall hepatic pedicle clamping time over 30 minutes long during the first stage.

In our Center, the morbidity rates after ALPPS are comparable to those of recent publications. Our study suggests that prolonged cycles of induction chemotherapy and long hepatic pedicle clamping times are associated with a worse prognosis with deleterious clinical outcomes.

**Molecular spectrum of KRAS/NRAS/BRAF in advanced colorectal cancer patients – one-year systematic review in a single tertiary centre**

Ana Gomes^1,3^, Paulo Teixeira^1^, Ângela Jesus^1^, Rui Caetano Oliveira^1,2,3^, Manuela Meruje^1^, Mário Rui Silva^1^, Maria Augusta Cipriano^1^

^*1*^*Pathology Department, Centro Hospitalar e Universitário de Coimbra, 3000–075, Coimbra, Portugal;*^*2*^*Biophysics Institute, Faculty of Medicine, University of Coimbra, Portugal;*^*3*^*iCBR-Coimbra Institute for Clinical and Biomedical Research – Area of Environment, Genetics and Oncobiology (CIMAGO), Faculty of Medicine, University of Coimbra*

**Aims/Context:** Colorectal cancer (CRC) is a world health concern, with a highly incidence and mortality. On advanced stages of disease – stage III/IV, the detection of RAS and BRAF mutation is mandatory in order to assess therapeutic resistance and disease prognosis. Since 2019 that these mutations are detected at our institution – a tertiary hospital, by an Idylla cartridge system. Our objective is to assess the reality of CRC mutations with the new system.

**Methods:** Retrospective analysis of RAS and BRAF mutations on CRC, with clinical and pathological correlation, over one-year period – 2019.

**Results:** We performed a total of 123 tests, 81 males (65.9%) and 42 females (34.1%) with a median age of 65 ± 11.14years-old (36–87) and found mutations in 74 patients (60.2%) – 48 males (64.9%) and 26 females (35.1%); 17 patients (13.8%) were over 75 years-old. There was a slight prevalence for left sided CRC – 64.2%. KRAS mutations were the most prevalent – 59 (79.7%), followed by BRAF – 12 patients (16.2%) and NRAS in 3 patients (4.1%).

There was an association between age, equal or superior to 76 years-old, and KRAS mutations (p = 0.042) and BRAF mutations and right-sided located CRC (p = 0.005) and defect on DNA mismatch repair proteins (p = 0.009).

**Conclusions:** Mutational testing is fundamental in CRC risk stratification and as a crucial theranostic tool. Our results with Idylla cartridge system seem solid and are comparable to what is stated in the literature, namely in what concerns BRAF mutations. The higher prevalence of KRAS mutations in patients older than 76 years-old is worthy of interest, because only patients up to 75 years-old are elected for routine testing. This may suggest that patient status as fit for therapy should be the driver for mutational testing.

**Application of the Paris System for Reporting Urinary Cytology at a tertiary hospital**

Anabela Pereira^1^, Rui Almeida^1,4^, Rui Caetano Oliveira^1,2,3^, Vítor Sousa^1,4^, Edgar Tavares-Silva^3,4,5^, Arnaldo Figueiredo^3,4,5,6^, Graça Fernandes^1^, Maria Augusta Cipriano^1^

^*1*^*Pathology Department, Centro Hospitalar e Universitário de Coimbra, Portugal;*^*2*^*Biophysics Institute, Faculty of Medicine, University of Coimbra, Portugal;*^*3*^*iCBR-Coimbra Institute for Clinical and Biomedical Research – Area of Environment, Genetics and Oncobiology (CIMAGO), Faculty of Medicine, University of Coimbra and CIBB - Center for Innovative Biomedicine and Biotechnology, University of Coimbra, Portugal;*^*4*^*Institute of Pathological Anatomy, Faculty of Medicine, University of Coimbra, Portugal;*^*5*^*Urology and Renal Transplantation Department, Centro Hospitalar e Universitário de Coimbra, Portugal;*^*6*^*CACC-Clinical Academic Center of Coimbra, Portugal;*

**Aims/Context:** Urothelial carcinoma accounts for 10% of urinary tract neoplasms, mostly in the bladder (90%). In 2014 was the eight more common cancer in Portugal. The main purpose of urinary cytology (UC) is the identification of high-grade urothelial carcinoma. Recently the Paris System Working Group elaborated a reporting system (PRS) with seven diagnostic categories: 1) Nondiagnostic (ND); 2) Negative for High-Grade Urothelial Carcinoma (NHGUC); 3) Atypical Urothelial Cells (AUC); 4) Suspicious for High-Grade Urothelial Carcinoma (SHUC), 5) High-Grade Urothelial Carcinoma (HGUC); 6) Low-Grade Urothelial Neoplasia (LGUN) and 7) Other Malignancies Primary and Metastatic and Miscellaneous Lesions (other).

We intend to retrospectively apply the PRS classification to the UC reports and establish a correlation with subsequent cytology and histology.

**Methods:** Studied all patients that underwent urinary cytology (UC) for the first time, in 2014 at our institution. We collected clinical data and the subsequent cytological/histological examination, applying the PRS. Correlation of cytology with histology was performed whenever there was a biopsy up to six months after cytology.

**Results:** 614 patients, with a median of 67 ± 16.2 years-old (15–97), 234 female (38.1%) and 380 male (61.9), were identified with the following results: 20 ND (3.3%), 346 NHGUC (56.4%), 115 AUC (18.7%), 6 SHUC (1%), 24 HUC (3.9%), 102 LGUC (16.6%) and 1 other (0.2%). 268 (43.6%) of patients had at least 2 UC; 114 patients (18.6%) had a histology report within 6 months after UC.

After a NHGUC result, 51.4% had a second UC with a NHGUC result, 21.8% with AUC and only 24.6% with LGUC; patients with AUC result reported on the second UC mostly a NHGUC result – 59.5%. When compared the first UC with the last 78.8% of patients with NHGUC had a NHGUC/AUC and only 15% of patients with AUC on first UC had a low or high-grade tumour. Comparison with histology showed a sensibility of 75.7% and a specificity of 59.1%.

**Conclusions:** Standardization of UC with PRS allows results reproducibility and a good sensibility towards histology. Its routine application should result in improvement on patients follow-up.

**Efficacy of cold atmospheric plasma against bladder cancer, *in vitro* studies**

Eurico Pereira^1,2,3^, Edgar Tavares-da-Silva^1,2,3,^ Ana R. Neves^1,2^, Ana S. Pires^1,2,3^, Catarina Guilherme^1,2,3,6^, Inês Marques^1,2,3,4,5^, Ana M. Abrantes^1,2,3,^ Rafael Silva-Teixeira^1,2^, Arnaldo Figueiredo^1,2,3,4^, Maria Filomena Botelho^1,2,3^

^*1*^* Biophysics Institute, Faculty of Medicine, University of Coimbra, Portugal;*^*2*^* CACC-Clinical Academic Center of Coimbra, Portugal;*^*3*^* iCBR-Coimbra Institute for Clinical and Biomedical Research – Area of Environment, Genetics and Oncobiology (CIMAGO), Faculty of Medicine, University of Coimbra and CIBB - Center for Innovative Biomedicine and Biotechnology, University of Coimbra, Portugal;*^*4*^*Centro Hospitalar e Universitário de Coimbra, Department of Urology and Renal Transplantation, Coimbra, Portugal;*^*5*^* Faculty of Pharmacy, University of Coimbra, Portugal;*^*6*^* Faculty of Medicine, University of Porto, Portugal*

**Aims/Contex:** Bladder cancer (BC) is the 6^th^ with the highest incidence and the 8^th^ with the highest mortality in Portugal. There are gaps in the development of new therapies for BC and drugs available in clinical use do not have the desired efficacy and show side effects. Cold Atmospheric Plasma (CAP) is a promising area that can have significant antitumor effects on cancer tissue, with cell destruction rates of over 90%, sparing normal tissue. CAP is a partially ionized gas, it is produced at a temperature below 40°C, and therefore does not induce thermal injury. Its biological effects and mechanisms of action result from interactions between plasma components with specific structural cellular elements, affecting tumor cells mechanisms. Our aim is to determine the efficacy of CAP treatment in BC cell lines.

**Methods:** An electronic device able to generate a high voltage discharge was designed by our group to create CAP. Two BC cell lines, HT1376 (grade III) and TCCSUP (grade IV) were used. Both cell lines were submitted to CAP treatment, which was generated in open air, 2 mm above the surface of the cell culture medium, during short periods of time (15, 30, 60, 90 and 120 seconds). 24 hours after treatment, metabolic activity was assessed by MTT assay and protein content by SRB assay, to indirectly evaluate cell proliferation. Also, 4, 6 and 24 hours after treatment, reactive oxygen species (ROS) and antioxidant defenses were measured.

**Results:** After CAP treatment, cell proliferation decreased in both cell lines dependent on the exposure time, however, TCCSUP cell line seems to be more sensitive to the therapy. In terms of oxidative stress for both cell lines, results demonstrate a tendency to increased intracellular production of radical superoxide and a decreased intracellular production of peroxides, while no statistical differences were observed in GSH levels.

**Conclusions:** The results obtained so far show that CAP has an anti-proliferative effect even after short periods of exposure. These outcomes encourage further studies and reveal that intravesical therapy with CAP is a promising therapeutic approach against BC.

Funding: FCT Portugal (SFRH/BD/116794/2016); PEst-UID/NEU/04539/2013 and FEDER-COMPETE (FCOMP-01-0124-FEDER-028417 and POCI-01-0145-FEDER-007440),

**Human amniotic membrane extract as an approach to hepatocellular carcinoma: effects of protein fractionation and lipids isolation**

Ricardo Jorge Teixo^1,2,3^, Mafalda Laranjo^1,2,3^, Maria João Carvalho^1,2,3,4^, Beatriz Serambeque^1,2,3^, Patrícia de Oliveira^1,2,3^, Paulo Moura^4^, Ana Margarida Abrantes^1,2,3^, Pedro Domingues^5^, Maria Filomena Botelho^1,2,3^

^*1*^*Biophysics Institute, Faculty of Medicine, University of Coimbra, Portugal;*^*2*^*iCBR-Coimbra Institute for Clinical and Biomedical Research – Area of Environment, Genetics and Oncobiology (CIMAGO), Faculty of Medicine, University of Coimbra and CIBB - Center for Innovative Biomedicine and Biotechnology, University of Coimbra, Portugal;*^*3*^*CACC-Clinical Academic Center of Coimbra, Coimbra, Portugal;*^*4*^*Gynecology Service, Coimbra Hospital and University Centre, Coimbra, Portugal;*^*5*^*Centro de Espetrometria de Massa, Departamento de Química & QOPNA& LAQV-REQUIMTE, University of Aveiro, Aveiro, Portugal*

**Aims/Context:** Hepatocellular carcinoma (HCC) is the most common liver cancer, with high incidence and mortality rates worldwide. HCC is mostly diagnosed at late stages which leads to a poor diagnosis and treatment efficiency. Human amniotic membrane (hAM) presents immunoregulatory, anti-angiogenic and pro-apoptotic activity. Our team previously showed that total human amniotic membrane extract (hAME) leads to decreased viability and increased cell death on HCC cells. With this work we aimed to evaluate the effect of different hAME fractions on HCC cells.

**Methods:** Having previously verified that hAME comprises a complex protein mixture, fractionation was performed considering solubility, through ammonium sulphate (AS) precipitation. Sequential precipitation was performed by adding 10%, 25% and 50% AS to hAME, on ice for 15 min, followed by centrifugation at 14000 G, 15 min. Precipitated fractions (10P, 25P and 50P) were resuspended on PBS and soluble fractions (10S, 25S and 50S) were submitted to salting out with PBS by centrifugation at 4000G, during 60 min on centrifuge tubes with membrane pore of 30 kDa cutoff. hAME was submitted to lipid isolation by the Folch method, where lipids are isolated from a 2:1 chloroform:methanol mixture, with two lipid extraction steps and solvent evaporation on vacuum. HCC cell lines HepG2, Hep3B and HuH7.sil were incubated total hAME, fractions and isolated lipids with a concentration of 1 μg/μL (protein or lipids accordingly), for 72 h. Metabolic activity was accessed by MTT assay.

**Results:** Our results demonstrated that incubation of HCC cells with hAME induced a decrease on metabolic activity, compared to control (HepG2: 57.07 ± 9.26; Hep3B: 50.66 ± 9.78; HuH7.sil: 58.16 ± 6.05). Incubation with fractions obtained from AS precipitation induced a lower metabolic activity compared to incubation with total hAME. Values of metabolic activity varied from 2 to 30%, depending on HCC cell line. Metabolic activity of HCC cells incubated to isolated lipids decreased compared to control cells and total hAME.

**Conclusions:** These findings indicate that both proteins and lipids could have an important role on the main anti-tumor activity of hAME.

Funding: FCT Portugal (SFRH/BD/116794/2016); PEst-UID/NEU/04539/2013 and FEDER-COMPETE (FCOMP-01-0124-FEDER-028417 and POCI-01-0145-FEDER-007440), FIS-INFARMED (FIS-2015-01-Onc-20150630-120)

**Establishment of a 3D cell culture model of metastatic prostate cancer to evaluate the effects of Radium-223**

Nuno Tiago Tavares^1,2,3^, Inês Alexandra Marques^1,2,3,4^, Ana Salomé Pires^1,2,3^, Paulo Teixeira^5^, Sandra Silva^1^, Edgar Tavares-Silva^6^, Francisco Caramelo^3,7^, Gracinda Costa^8^, Arnaldo Figueiredo^6^, Ana Margarida Abrantes^1,2,3^, Maria Filomena Botelho^1,2,3^

^*1*^*Biophysics Institute, Faculty of Medicine, University of Coimbra, Coimbra, Portugal;*^*2*^*Clinical Academic Center of Coimbra, Coimbra, Portugal;*^*3*^* iCBR-Coimbra Institute for Clinical and Biomedical Research – Area of Environment, Genetics and Oncobiology (CIMAGO), Faculty of Medicine, University of Coimbra and CIBB - Center for Innovative Biomedicine and Biotechnology, University of Coimbra, Portugal;*^*4*^*Faculty of Pharmacy, University of Coimbra, Portugal;*^*5*^*Department of Pathology, Centro Hospitalar e Universitário de Coimbra, Portugal;*^*6*^*Department of Urology and Renal Transplantation, Centro Hospital e Universitário de Coimbra, Portugal;*^*7*^*Laboratory of Biostatistics and Medical Informatics, Faculty of Medicine, University of Coimbra, Portugal;*^*8*^*Department of Nuclear Medicine, Centro Hospitalar e Universitário de Coimbra, Portugal.*

**Context/Objectives:** Nowadays, metastatic castration-resistant prostate cancer is a stage of the disease that still presents a high mortality rate. One of few therapeutic options is the Radium-223, an alpha particle emitter radiopharmaceutical, however the knowledge regarding its targets and its effect in the metastatic niche is present as a limitation. Thus, the main goal was to optimize and characterize a three-dimensional culture of metastatic prostate cancer cells, and then assess the effects of the Radium-223 on the model.

**Methods:** The magnetic levitation method was used to form three-dimensional spheroids of PC3. Then, histochemical staining, to study spheroid structure and viability, immunocytochemistry for protein expression, and flow cytometry to learn about the culture's viability and cell death pathways were performed. The effects of the Radium-223 irradiation were then studied by the SRB and Alamar Blue assays.

**Results:** The PC3 3D structures displayed a spherical conformation and presented extensive necrotic and apoptotic zones, since the size of the spheroid was in the order of mm2. Besides, the spheroids also exhibited different expression in some key proteins when compared with control cells cultured in monolayer, an important fact when testing cancer therapeutics.

After the irradiation of the spheroids with Radium-223, all doses tested presented a decrease compared to the control whether it was in total protein content or cell viability, however, at lowest values when compared with monolayer cells. This can be because the three-dimensional structures are closer to mimicking the in vivo scenario, and so the cytotoxicity of the Radium-223 is decreased. Also, as alpha particles have low penetration ranges and the spheroid has a large size, the radiopharmaceutical might not be properly entering the three-dimensional structure.

**Conclusions:** Thereby, with this work it was possible to conclude that, as expected, the Radium-223 acts differently when tested in monolayer and in three dimensional cultures, as shown by the primary results obtained.

Funding: FCT Portugal (SFRH/BD/116794/2016); PEst-UID/NEU/04539/2013 and FEDER-COMPETE (FCOMP-01-0124-FEDER-028417 and POCI-01-0145-FEDER-007440) and SFRH/BD/136973/2018.

**Splenosis and Pancreatic Cancer: A Challenging Diagnosis**

Andreia Guimarães^1^, Henrique Alexandrino^1,2,3,4^, Ana Velez^1,2^, José Guilherme Tralhão^1,2,3,4^

^*1*^*General Surgery Department, Coimbra University Hospital Center, Coimbra, Portugal;*^*2*^*Faculty of Medicine, University of Coimbra, Portugal;*^*3*^* iCBR-Coimbra Institute for Clinical and Biomedical Research – Area of Environment, Genetics and Oncobiology (CIMAGO), Faculty of Medicine, University of Coimbra and CIBB - Center for Innovative Biomedicine and Biotechnology, University of Coimbra, Portugal*^*4*^*CACC-Clinical Academic Center of Coimbra, Portugal;*

**Background:** Splenosis is a rare benign condition resulting from ectopic splenic tissue sedding after splenic surgery or trauma. It is usually asymptomatic and incidentally found and can mimic other pathologic entities.

We report a case of an incidental asymptomatic disseminated pancreatic cancer hidden by splenosis.

**Methods:** A 64-years-old male was evaluated six years prior for constipation. The patient history included splenic trauma with splenectomy and direct family with colon and gastric cancer. Physical exam was unremarkable. Complete laboratory workup, upper and lower endoscopic exams were normal. Computerized tomography revealed abdominal nodules, suggesting splenosis, and a heterogeneous pancreatic tail, remaining stable in routinely repeated exams. He then presented with bilateral extremities paresthesias. A paraneoplastic presentation was suggested. After clinical and workup reassessment, an increase in cancer antigen 19.9 was found and Magnetic Resonance Imaging revealed an expansive lesion in the pancreatic tail in close relation to splenosis foci. A few months later CT-scan was repeated, sustaining an uncharacteristic pancreatic tail alteration. MRI revealed a pancreatic lesion growth, splenic nodules invasion and a hepatic nodule. A ^99^Technetium-labelled red blood cell scintigraphy was performed revealing splenosis.

**Results:** Due to pancreatic malignancy suspicion surgery was performed revealing peritoneal nodules, a liver nodule and a retroperitoneal mass related do the pancreatic tail. Biopsies were performed and frozen-section exam revealed adenocarcinoma. Histopathologic exam confirmed pancreatic adenocarcinoma metastasis and splenosis. Patient was discharged home 2 days after surgery with no complications. He is undergoing palliative chemotherapy with FOLFIRINOX.

**Conclusions:** Incidental imaging findings of splenosis foci can present as a confounding factor in patients with abdominal abnormalities and splenic trauma or surgery. Other diagnosis such as malignancy must be ruled out. The case emphasizes the need to carefully evaluate patients and imaging exams in order to prevent unnecessary operations or promptly undergo required procedures.

**Synthesis and antitumor evaluation of new steroidal oximes in several cancer cell lines**

Ana Rita Gomes^1,2,3^, Ana Salomé Pires^1,4,5^, Ana Margarida Abrantes^1,4,5^, Carla Varela^2,6^, Elisiário Tavares-da-Silva^2,6^, Maria Filomena Botelho^1,4,5^, Fernanda Maria Fernandes Roleira^2,6^

^*1*^*Biophysics Institute, Faculty of Medicine, University of Coimbra, Portugal;*^*2*^*Laboratory of Pharmaceutical Chemistry, Faculty of Pharmacy, University of Coimbra, Portugal;*^*3*^*Faculty of Sciences and Technology, University of Coimbra, Portugal;*^*4*^*CACC-Clinical Academic Center of Coimbra, Portugal;*^*5*^*iCBR-Coimbra Institute for Clinical and Biomedical Research – Area of Environment, Genetics and Oncobiology (CIMAGO), Faculty of Medicine, University of Coimbra and CIBB Center for Innovative Biomedicine and Biotechnology, Coimbra, Portugal;*^*6*^*CIEPQPF Centre for Chemical Processes Engineering and Forest Products, University of Coimbra, Portugal*

**Introduction:** Cancer is a worldwide disease, causing numerous deaths every year. Steroidal compounds were proven to be efficient against several types of cancer. Oximes are also a structural feature frequently associated with anticancer activity. Thus, our aim is to combine this feature with the steroidal backbone by synthesizing steroidal oximes and evaluating their activity against several cancer cell lines, ultimately to find new anticancer agents with fewer side effects.

**Methods:** The compounds 5α-androst-3-en-17-one oxime (**3,4–OLOX**) and androst-4-en-17-one oxime (**4,5–OLOX**) were synthesized and their cytotoxicity evaluated in four human cancer cell lines, namely, colorectal adenocarcinoma (WiDr), non-small lung cancer (H1299), prostate cancer (PC3) and liver hepatocellular carcinoma (HepG2). We used MTT assay to assess cell proliferation, flow cytometry to evaluate viability and cell death and fluorescence to measure ROS production, after treatment with our compounds. A normal colon cell line was also used (CCD841 CoN). Hemocompatibility was assessed by hemoglobin release measurement.

**Results:** The most effective compound in all cell lines was **3,4–OLOX**, particularly in WiDr and PC3 cells, demonstrating the best IC_50_ results (9.1 μM and 13.8 μM, respectively). **4,5–OLOX** also showed promising results in the same cell lines (IC_50_ = 14.5 μM in PC3 and IC_50_ = 16.1 μM in WiDr). Additional studies with CCD841 CoN cells revealed the selectivity of **3,4–OLOX** and **4,5–OLOX** towards WiDr cancer cells. **3,4–OLOX** and **4,5–OLOX** induced a decrease in cell viability accompanied by an increase of cell death, mainly by apoptosis or apoptosis/necrosis. **3,4–OLOX** and **4,5–OLOX** increases ROS production (intracellular peroxides and superoxide anion) in all treated cells compared to non-treated cells. This might indicate ROS production as a possible mechanism of action. Both compounds did not induce hemoglobin release with both tested concentrations, 10 and 75 μM.

**Conclusion:** Results suggest that **3,4–OLOX** and **4,5–OLOX** might have an anticancer effect mediated by apoptosis/necroptosis and by ROS production, which encourages further studies. Compounds also proved to be safe for I.V. administration.

Funding: FCT Portugal (SFRH/BD/116794/2016); PEst-UID/NEU/04539/2013 and FEDER-COMPETE (FCOMP-01-0124-FEDER-028417 and POCI-01-0145-FEDER-007440),

**Corroles as Photosensitizers for Photodynamic therapy**

João Braz^1,2^, Mafalda Laranjo^1,3^, Susana Lopes^2^, Marta Piñero^2^, Maria Filomena Botelho^1,3^, Teresa P. Melo^2^

^*1*^*Biophysics Institute, Faculty of Medicine, University of Coimbra, Portugal;*^*2*^*CQC and Department of Chemistry, University of Coimbra, Portugal;*^*3*^*iCBR-Coimbra Institute for Clinical and Biomedical Research – Area of Environment, Genetics and Oncobiology (CIMAGO), Faculty of Medicine, University of Coimbra and CIBB Center for Innovative Biomedicine and Biotechnology, Coimbra, Portugal*

**Aims/Context:** Corrole is a ring-contracted tetrapyrrolic macrocycle of the porphyrin family. Over the last decades, this smaller analogue of porphyrin has received much attention in the field of antitumor drugs development, particularly in photodynamic therapy (PDT). PDT consists of a light-activated chemical reaction used to selectively destroy tissues, through the generation of singlet oxygen and other reactive oxygen species (ROS).

**Methods:** In this work, a new chemical library of corroles was synthetized through a bis-hetero-Diels-Alder reaction between an oxime and an dipyrromethane, followed from a oxidation with 2,3-dichloro-5,6-dicyano-1,4-benzoquinone (DDQ). *In vitro* studies of these corroles based photodynamic therapy were also carried out in lung cancer cell lines. For that, A549 and H1299 were growth in used Dulbecco's Modified Eagle's Medium (DMEM) supplemented with 10% and 5% fetal bovine serum respectively, 100 μM sodium pyruvate and 1% antibiotic, and maintained at 37°C in an humidified atmosphere of 5% CO_2_. Then, photosensitizers were solubilized in dimethylsulfoxide (DMSO) and administrated to cells in different concentrations ranged from 50 nM to 10 μM, and cells incubated for 24 hours. After this time, two different experiences were performed, in order to test photocytotoxicity and cytotoxicity. For photocytotoxicity assays, the cultures were washed with PBS, irradiated with a photon flux of 7.5 mW/cm^2^, to a total of 10 J, and incubated for 24 hours before MTT assay. For cytotoxicity assays, the cultures were washed with PBS and incubated for 24 hours before MTT assay.

**Results:** The preliminary MTT assays of these PS showed that, when under irradiation, the concentration value for the 50% inhibition of the metabolic activity (IC50) of some of these PS was in the nanomolar range in all cell lines tested. Furthermore, all corroles showed no dark citotoxicity in both cell lines.

**Conclusion:** The current results, with IC50 values in the nanomolar range, point to a promising therapeutic effect, and support further studies.

Funding: FCT Portugal (SFRH/BD/116794/2016); PEst-UID/NEU/04539/2013 and FEDER-COMPETE (FCOMP-01-0124-FEDER-028417 and POCI-01-0145-FEDER-007440) and by FEDER through COMPETE 2020 (project no. POCI-01-0145FEDER-PTDC/QEQ-MED/0262/2014), PT 2020/CENTRO 2020 (project no. CENTRO-01-0145-FEDER-000014/ MATIS). CQC is supported via project no. POCI-01-0145-FEDER-007630.

**Plasma based therapies in cancer treatment**

Silva-Teixeira R^1,2^, Laranjo M^1,2,3^, Almeida-Ferreira C^1^, Lopes B^1^, Abrantes AM^1,2,3^, Caramelo F^1,4^, Botelho MF^1,2,3^

^*1*^*Biophysics Institute, Faculty of Medicine, University of Coimbra, Portugal;*^*2*^*iCBR-Coimbra Institute for Clinical and Biomedical Research – Area of Environment, Genetics and Oncobiology (CIMAGO), Faculty of Medicine, University of Coimbra and CIBB Center for Innovative Biomedicine and Biotechnology, Coimbra, Portugal;*^*3*^*CACC-Clinical Academic Center of Coimbra, Portugal;*^*4*^*Laboratory of Biostatistics and Medical Informatics, Faculty of Medicine, University of Coimbra, Portugal*

**Aim/ Context:** A new therapy based on plasma, the fourth state of matter, has recently raised the medical community's attention by circumventing many undesirable effects of old anticancer treatments. The aim of this work was to evaluate the effect, selectivity and mechanisms of action of cold atmospheric plasma (CAP) in a human retinoblastoma cell line.

**Methods:** An electronic device was designed to generate CAP, in open air, 2 mm above seeded cell cultures. Timely-resolved output voltage measurement, emission spectroscopy and quantification of reactive species (RS) in plasma activated media (PAM) were performed to characterize the physical and chemical properties of plasma. In order to evaluate the cytotoxicity and selectivity of CAP, the metabolic activity of similarly treated Y79, fibroblasts HFF1 and retinal RPE-D407 cells was assessed. Clonogenic assay screened for survival. Apoptosis detection, analysis of mitochondrial membrane potential (MMP) and cell morphology were performed to clarify the type of cell death. Cell cycle distribution and genotoxic effects were evaluated by flow cytometry and comet assay, respectively. Intracellular RS and oxidative defenses were quantified. In order to evaluate indirect effects of plasma treated liquids, cells were incubated with PAM and conditionated media (CM). Finally, antiangiogenic effects were tested with aortic ring assay.

**Results:** After 60 s of direct CAP treatment, the metabolic activity of retinoblastoma cells decreased more than 50%, mainly due to apoptosis, while HFF1 and RPE-D407 remained viable. Similar results were obtained with indirect treatment (PAM and CM). Cell survival was reduced and cells accumulated in S and G2/M phases, however, no DNA strand breaks were detected. Regarding RS, plasma increased extracellular and intracellular concentrations of peroxides and nitric oxide, despite no activation of antioxidative defenses and lack of success in reverting cytotoxicity with RS inhibitors. Additionally, maximal antiangiogenic effects were obtained with 60 s of plasma exposure.

**Conclusion:** Direct and indirect treatment with CAP might be a selective therapy with the potential to target tumor cells and supporting microenvironment.

Funding: FCT Portugal (SFRH/BD/116794/2016); PEst-UID/NEU/04539/2013 and FEDER-COMPETE (FCOMP-01-0124-FEDER-028417 and POCI-01-0145-FEDER-007440).

**Establishment of primary culture of cat intraocular melanoma**

Tarcísio Guimarães^1,2,3∗^, Karla Cardoso^2,3^, Nuno Alexandre^4^, Rui Oliveira^2,3^, Maria Filomena Botelho^2,3,5^, Mafalda Laranjo^2,3,5^

^*1*^*Institute of Agrarian and Environmental Environmental Sciences (ICAAM), University of Évora, Portugal;*^*2*^*Institute of Biophysics, Faculty of Medicine, University of Coimbra, Portugal;*^*3*^*iCBR-Coimbra Institute for Clinical and Biomedical Research – Area of Environment, Genetics and Oncobiology (CIMAGO), Faculty of Medicine, University of Coimbra and CIBB Center for Innovative Biomedicine and Biotechnology, Coimbra, Portugal*^*4*^*Department of Veterinary Medicine, University of Évora, Portugal.*^*5*^*CACC-Clinical Academic Center of Coimbra, Portugal;*

**Aim/ Context:** Culture of animal and human cells are widely employed in biomedical research, as experimental models of living organisms. The objective of this investigation was to develop a method to obtain primary cell culture of intraocular neoplasms from company animals, to be further used in the study of novel anti-cancer therapies.

**Methods:** The tumor samples were obtained from two cats (*Felis catus*), with a history of intraocular neoformation and indication for enucleation. The donor globes were dissected and a small fraction of the affected tissues were collected for cell culture, being the rest the eye globe subjected to histopathological analysis. The collected tissues were dissected into pieces of approximately 1 mm in Petri dishes, while immersed in PBS with antibiotics. Two culture protocols were tested: the enzymatic digestion and the explant culture of the digested tumor fragments. The 1 mm tumor fragments were incubated with Trypsin in DMEM culture medium for 60 min with mechanical stirring at 37 ° C. The digestion medium was collected, centrifuged at 2000 xg and the sedimentation fraction (cell pellet) was collected. These procedure was repeated three times and the cells collected were cultured in adherent culture conditions, with F12 medium supplemented with 10% of fetal bovine serum, 1 mg/mL of cholera toxin and antibiotics. The tumor fragments collected after the enzymatic digestion process were evenly distributed in a culture dish and maintained with F12 culture medium. The biological material was kept in an incubator at 37 ° C, 5% CO^2^ and 90% humidity. Cell culture medium was renewed every two to three days and the cultures were monitored by microscopy.

**Results:** Histopathological results revealed characteristics of neuroectodermal and melanocytic origin. It was possible to obtain high cellularity, and cells with replication capacity in culture. Considering the samples described, explant culture proved to be the most suitable technique for intraocular tissues.

**Conclusion:** The present work allowed to obtain a primary cell culture of cat intraocular neoplasms, which suggests the possibility of using these cells in further studies, such as the evaluation of anticancer therapies.

Funding: FCT Portugal (SFRH/BD/116794/2016); PEst-UID/NEU/04539/2013 and FEDER-COMPETE (FCOMP-01-0124-FEDER-028417 and POCI-01-0145-FEDER-007440) and SFRH/BD/139319/2018.

**Evaluation of cell proliferation in breast cancer cell lines by the MTT assay**

Karla Cardoso^1,2^, Tarcísio Guimarães^1,2^, Fernando Capela e Silva^3^, Nelson Pereira^4^, Bruno Nascimento^4^, Marta Pinheiro^4^, Teresa Pinho e Melo^4^, Mafalda Laranjo^2,5,6^, Maria Filomena Botelho^2,5,6^

^*1*^*Institute of Agrarian and Environmental Environmental Sciences (ICAAM), University of Évora, Portugal;*^*2*^*Institute of Biophysics, Faculty of Medicine, University of Coimbra, Portugal,*^*3*^*Department of Biology, University of Évora, Portugal;*^*4*^*CQC and Department of Chemistry, University of Coimbra, Portugal,*^*5*^*iCBR-Coimbra Institute for Clinical and Biomedical Research – Area of Environment, Genetics and Oncobiology (CIMAGO), Faculty of Medicine, University of Coimbra and CIBB Center for Innovative Biomedicine and Biotechnology, Coimbra, Portugal;*^*6*^*CACC-Clinical Academic Center of Coimbra, Portugal;*

**Aim/ Context:** Photodynamic therapy (PDT) is a promising therapeutic modality in medicine and veterinary medicine. Due to the clinical results and the simplicity of the technique, PDT can be an effective alternative for the treatment of breast tumors in dog's female. The aim of the work was to evaluate the effect of newly developed ring-fused 5, 15-diphenylchlorins on cell proliferation by testing in human breast and dogs’ female mammary carcinoma (CM) cells.

**Methods:** The cell lines REM134, MCF7 and HCC1806 were subjected to the photosensitizers at concentrations between 1nM-1 μM for 24 hours. The culture medium was renewed, and the cell cultures irradiated (ƛ > 570 nm) with 10J (7.5mW/cm^2^). The cytotoxicity assessment was performed 24 hours later using the MTT assay. For this, plates were washed and incubated with MTT solution during 4 hours, at 37°C. Formazan crystals were solubilized with hydrochloric acid in isopropanol and absorbance read at 570 nm with a 620 nm reference filter.

**Results:** The dihydroxymethyl ring-fused chlorin showed the highest activity at the lowest concentration tested of 25 nM in the HCC1806 line. Moreover, in these cells the dicarboxylic acid ring-fused chlorin also showed promising photodynamic activity with concentrations equal or superior to 100 nM. Preliminary results in the REM134 cells, point to the interesting outcome of the dicarboxylic acid ring-fused chlorin. This chlorin also showed satisfactory results in the MCF7 cells, in concentrations equal or superior to 100 nM, being more efficient than the dicarboxylic acid Pt(II) ring-fused coumpond. The dihydroxymethyl Pt(II) ring-fused chlorin was not as effective as dihydroxymethyl ring-fused, which showed cell death results at a concentration of 50 nM. Thus, the dihydroxymethyl ring-fused chlorin was the molecule that represented the greatest performance in PDT in the MCF7 line.

**Conclusion:** The photosensitizers used are promising, particularly the dihydroxymethyl ring-fused chlorin and the dicarboxylic acid ring-fused chlorin. This approach might become an option in the treatment of breast carcinoma in medicine and veterinary medicine.

Funding: FCT Portugal (SFRH/BD/116794/2016); PEst-UID/NEU/04539/2013 and FEDER-COMPETE (FCOMP-01-0124-FEDER-028417 and POCI-01-0145-FEDER-007440).

**Lynch Syndrome molecular profile in Liquid Biopsy**

Ana Oliveira,^1^ Luís M Nogueira,^2^ Carolina Ribeiro,^2^ Rui Almeida,^3^ Margarida Abrantes^3,5,6^, José G Tralhão^1,3,5,6^, Henriqueta Coimbra Silva^2,3,6^

^*1*^*Serviço de Cirurgia Geral, Centro Hospitalar e Universitário de Coimbra, Portugal;*^*2*^* Institute of Medical Genetics/UCGenomics, Faculty of Medicine, University of Coimbra;*^*3*^*iCBR-Coimbra Institute for Clinical and Biomedical Research – Area of Environment, Genetics and Oncobiology (CIMAGO), Faculty of Medicine, University of Coimbra and CIBB Center for Innovative Biomedicine and Biotechnology, Coimbra, Portugal;*^*4*^*Serviço de Anatomia Patológica, Centro Hospitalar e Universitário de Coimbra, Portugal;*^*5*^*Instituto de Biofísica, Faculdade de Medicina, Universidade de Coimbra, Portugal;*^*6*^*CACC-Clinical Academic Center of Coimbra, Portugal;*

**Introduction**: Lynch syndrome, the main cause of hereditary colorectal cancer (CRC), is an autosomal dominant disorder caused by loss of function of mismatch repair (MMR) proteins, resulting in poor repair of DNA replication errors. Lynch syndrome tumors characteristically show a high mutation rate, accumulating somatic SNVs and indels. Despite the risk for family members and for multiple cancers in carriers, these tumours respond better to systemic therapies and have better prognosis. Liquid biopsy (LB) is emerging as a useful tool in the management of CRC patients as it allows a better assessment of cancer molecular heterogeneity.

**Aim:** We aim to verify if LB associated with New Generation Sequencing (NGS) technology can differentiate Lynch syndrome from other CRCs

**Methods:** Six CRC patients were studied. One of the patients, a 39 year old male, tumor immunohistochemistry showed loss of expression of MSH2 and MSH6, suggesting a Lynch Syndrome. Peripheral blood sample (5–7 ml) in EDTA tubes was processed for plasma collection within one hour. Cell-free DNA (cfDNA) was extracted from 2.5 -3.5 ml of plasma using MagMax cfDNA isolation Kit. Molecular profile was performed with Oncomine Colon cfDNA Assay. Ion Chef System and Ion 530 Kit-Chef were used for template preparation. A 50pM library pool containing six samples was applied in Ion 530 Chip and sequenced on Ion Gene Studio S5 Plus platform (Thermo Fisher Scientific). For variant analysis, Torrent Variant Caller plugin, Torrent Suite Software v5.2.1 and Ion Reporter online tool were used (Thermo Fisher Scientific).

**Results:** Plasma cfDNA concentration varied from 0.6 to 15.7 ng/μl. Using a cfDNA input ranging between 8 to 31 ng, limits of detection (LoD) from 1.25 to 0.1% were achieved. Driver mutations in seven genes, but not in BRAF, were identified in the LB of the patient suggestive of Lynch Syndrome. KRAS actionable mutations were detected in five patients, as well as driver mutations in three or more genes, the most frequent being KRAS, PIK3CA and APC.

**Conclusion:** LB captured the enriched mutation profile of Lynch patient, suggesting it may be useful for diagnosis when no primary tumor is available.

**Evaluating gut microbiota and inflammation after obesity surgery using Next-Generation Sequencing and mRNA identification**

André Lázaro^1,4^, Igor Tiago^2^, Luís M Nogueira^3^, Carolina Ribeiro^3^, António Veríssimo^2^, Fernando Jesus Regateiro^3^, Fernando J Oliveira^1,5^, Henriqueta C Silva^3,4,5^

^*1*^*General Surgery Unit, Surgical Treatment for Obesity Unit, Centro Hospitalar e Universitário de Coimbra, Portugal and Faculty of Medicine, University of Coimbra, Portugal;*^*2*^* Centre for Functional Ecology (CFE), Department of Life Sciences, University of Coimbra, Coimbra, Portugal;*^*3*^*Institute of Medical Genetics/UCGenomics, Faculty of Medicine, University of Coimbra, Portugal;*^*4*^* iCBR-Coimbra Institute for Clinical and Biomedical Research – Area of Environment, Genetics and Oncobiology (CIMAGO), Faculty of Medicine, University of Coimbra, and CIBB Center for Innovative Biomedicine and Biotechnology, Coimbra, Portugal;*^*5*^*CACC-Clinical Academic Center of Coimbra, Portugal;*

**Introduction:** Obesity is a major healthcare problem. Metabolic syndrome plays a significant role in obesity's morbidity and mortality.

Obesity surgery is an effective treatment for morbid obesity and metabolic syndrome, its effects target weight loss and improvement of linked medical conditions.

Gut microbiota plays a significant role in pathogenesis of obesity, the gut microbiota-human interaction is done through our immune system (cells, cytokines).

This study aims to evaluate gut microbiota and inflammation after obesity surgery, providing insight on how this interaction is made.

**Methods:** Prospective study including 24 patients, submitted to obesity surgery, 13 sleeve gastrectomy, 11 gastric bypass.

Patients were evaluated before and 6 months after surgery: antropometric data collection, peripheral blood sampling for biochemical analysis and inflammation evaluation and fecal sampling for gut microbiota identification.

Inflammation evaluated pro-inflammatory mRNA linked to bacterial immune response using SALSA MLPA KIT R009mRNA (MRC-Holland).

Gut microbiota evalution was done by sequencing V4 hypervariable region of 16S rRNA by NGS approach in MiSeq (Illumina).

**Results:** There was improvement of conditions related to obesity (p < 0,05): body mass index, waist circumference, insulin resistance, C-reactive protein, triglycerides, uric acid, and systolic blood pressure with no difference between bypass and sleeve.

Microbiota analysis revealed a high interindividual variability and a shift in phila representation after surgery, particularly for patients undergoing bypass.

Inflammatory mRNA analysis revealed statistically significant increase in some cytokines (Il-4,NF-kb IA,TNF,TNF receptor SF1A,Il-1b,IL-8,SCY A3 and 4,PDE 4B) after surgery.

**Conclusions:** Improvement of metabolic parameters was associated with a higher gut microbiota variability.

C-reactive protein was decreased after surgery, however, some inflammatory mRNA's were significantly increased after surgery.

Further evaluation of gut microbiota's inferior taxonomic categories might provide useful data to identify possible implications for these findings.

**Retrospective study of Array-CGH results in patients with neurodevelopment disorders**

Gabriela Penas^1^, Susana I. Ferreira^1^, Luís M. Pires^1^, Mariana Val^1^, Nuno Lavoura^1^, Luísa Esteves^1^, Joana B. de Melo^1,2,3^, Fabiana Ramos^4^, Margarida Venâncio^4^, Isabel M. Carreira^1,2,3^

^*1*^* Cytogenetics and Genomics Laboratory, Institute of Cellular and Molecular Biology, Faculty of Medicine, University of Coimbra, Portugal;*^*2*^*iCBR-Coimbra Institute for Clinical and Biomedical Research – Area of Environment, Genetics and Oncobiology (CIMAGO), Faculty of Medicine, University of Coimbra and CIBB Center for Innovative Biomedicine and Biotechnology, Coimbra, Portugal,*^*3*^*CACC-Clinical Academic Center of Coimbra, Portugal; Serviço de Genética Médica, Hospital Pediátrico, Centro Hospitalar e Universitário de Coimbra, Portugal*

**Introduction:** Array comparative genomic hybridization (aCGH) is the best approach to test unexplained neurodevelopmental disorders (NDDs). This are neuropsychiatric disorders characterized by the impairment of motor, learning and communication functions. The copy number variations (CNVs) found with this technique may be a key to identify patterns of variants in NDDs and understand the role of some variants of unknown significance (VOUS) in these disorders.

**Materials and Methods:** We did a retrospective study (2017/2018) on 527 patients with a median age of 8 years, CI 95%: [1.417;34.404], diagnosed with one or more NDDs from the 9 considered, that were tested by 60K and 180K oligonucleotide aCGH. The distribution of the different variants in the genome was determined for all the NDDs in this study aiming to identify the most frequently altered chromosomic regions.

**Results:** The aCGH results showed 376 clinically relevant CNVs in 257 patients. Of those 44 were classified as pathogenic and 332 were classified as VOUS. CNVs were observed in all chromosomes with higher frequency in chromosomes X, 7, 6 and 15 (in this order). There were 1.7 more duplications than deletions. Deletions were mainly detected in chromosomes 7,16, X and 1 while for duplication chromosomes X, 15, 6 and 7 were the primarily observed. Positive aCGH results were detected in approximately 50% of the patients, ranging between 35% and 67% within the nine NDDs groups considered. Psychomotor developmental delay and intellectual disability were the phenotypes with the higher number of patients, while intellectual developmental disorder had the lowest number, which may explain the 35% of positive results found in this group.

**Conclusion:** A heterogeneous distribution of variants was observed and an evident pattern in the CNVs of the different phenotypes could not be established.

In the follow up of this study, statistical tests will be used to ascertain a statistical significance between CNVs’ classification or frequency and NDDs. Other relevant phenotypes may simultaneously be studied.

***FMR1* gene: from primary ovarian insufficiency in grandmothers to children with intellectual disability**

Susana Isabel Ferreira^1^, Luís Miguel Pires^1^, Nuno Lavoura^1^, Mariana Val^1^, Jorge Saraiva^2^, Lina Ramos^2^, Isabel Marques Carreira^1,3,4^

^*1*^*Cytogenetics and Genomics Laboratory, Institute of Cellular and Molecular Biology, Faculty of Medicine, University of Coimbra, Portugal;*^*2*^*Serviço de Genética Médica, Hospital Pediátrico, Centro Hospitalar e Universitário de Coimbra;*^*3*^*iCBR-Coimbra Institute for Clinical and Biomedical Research – Area of Environment, Genetics and Oncobiology (CIMAGO), Faculty of Medicine, University of Coimbra and CIBB Center for Innovative Biomedicine and Biotechnology, Coimbra, Portugal,*^*4*^*CACC-Clinical Academic Center of Coimbra, Portugal.*

**Context:** Fragile X mental retardation*-*1 gene *(FMR1)* can have a variable number of CGG repeats at the 5′ untranslated region, ranging up to 45 repeats in normal alleles, between 45–54 CGG repeats in intermediate alleles, 55–200 CGG repeats in premutations (PM) and above 200 CGG repeats in full mutations (FM). FM are usually hypermethylated, the gene is transcriptionally silenced and the fragile X mental retardation protein (FMRP) is absent, and, as consequence, FM carriers have intellectual disability, developmental delay, autism spectrum disorders, and characteristic craniofacial features associated with Fragile X Syndrome (FXS). Premutation allele carrier females have up to 20% increased risk of primary ovarian insufficiency (POI) and Fragile X-associated tremor/ataxia syndrome (FXTAS), and males have 40% increased risk of FXTAS.

**Material and methods:** In the last 10 years we received over 2200 samples for *FMR1* gene testing, whose objective was to determine the allelic form present. This can be done by conventional PCR (cPCR), with primers flanking the CGG repeat region or Triplet Repeat Primed PCR (TRP-PCR) that has an extra internal primer, and allows the discrimination between homozygous and full mutation carrier females. It also detects long range pre-mutation and full mutation alleles, missed by cPCR.

The great majority of samples, 77.7% were studied due to a phenotype suggestive of FXS, 4.72% due to secondary amenorrhea and the remaining due to infertility, relatives of patients with PM or FM alleles or phenotype suggestive of FXS. In prenatal, 22 samples were tested for *FMR1* gene.

**Results and Conclusion:** In the prenatal samples, we identified 22.7% fetuses with FM, (3♂,2♀), allowing the couple to make an informed decision for the outcome of the pregnancy, knowing the child would have FXS. Of the 104 females with POI, 7.7% revealed to be PM, similar to the literature reports. In the group of patients with phenotype suggestive of FXS, 8 males and 7 females revealed to have FM, an overall lower frequency compared to the literature but with higher proportion of females. Relatives of all FM and PM carriers were studied and the implications and risks explained.

**Monitoring cfDNA levels in oral cancer patients: a tool for diagnosis and follow-up? – Pilot study**

Ivana Martins^1^; Leonor Barroso^2^; Inês Tavares^1^; Luís M. Pires^1^, Francisco Marques^3^, Joana B. Melo^1,4,5^; Isabel M. Carreira^1,5,6^, Ilda P. Ribeiro^1,4,5^

^*1*^*Cytogenetics and Genomics Laboratory, Institute of Cellular and Molecular Biology, Faculty of Medicine, University of Coimbra, Portugal;*^*2*^*Maxillofacial Surgery Department, Coimbra Hospital and University Centre, CHUC, EPE, Coimbra, Portugal;*^*3*^*Department of Dentistry, Faculty of Medicine, University of Coimbra, Coimbra, Portugal;*^*4*^*iCBR-Coimbra Institute for Clinical and Biomedical Research – Area of Environment, Genetics and Oncobiology (CIMAGO), Faculdade de Medicina da Universidade de Coimbra, Portugal and CIBB Center for Innovative Biomedicine and Biotechnology, Coimbra, Portugal,*^*5*^*CACC-Clinical Academic Center of Coimbra, Portugal.*

**Objectives/Context:** Oral cancer (OC) ranks within the top 10 most common cancers, having low survival rates, mainly due to late diagnosis and high rates of recurrence. Liquid biopsies, consisting of the analysis of cancer-derived components, such as circulating tumor DNA (ctDNA), in biofluids has recently emerged as a potential complement or alternative to tissue biopsies for diagnosis and follow-up, in a variety of cancers. However, their clinical impact in OC is still limited. Hence, in this study we aimed to obtain clues on the utility of liquid biopsies in OC, quantifying cell-free DNA (cfDNA) levels in OC patients and controls, and monitoring cfDNA concentrations before and after treatment initiation.

**Methods:** 61 urine and plasma samples from 9 OC patients and 4 plasma samples from healthy controls were included in the study. Samples from OC patients were collected before and at several timepoints following treatment. cfDNA was extracted from all the samples and its concentration was determined by fluorometry.

**Results and Conclusions**: Baseline plasma cfDNA levels in controls and OC patients were relatively similar. After treatment initiation, all patients presented a spike in plasma cfDNA, which seemed to decrease gradually in following timepoints, being in accordance with the expected. Patients undergoing chemotherapy–radiotherapy presented higher cfDNA concentrations right after treatment, than those undergoing surgery. Urine cfDNA levels were very heterogeneous among patients and did not display the tendencies observed in plasma. Notably, three late-stage (IV) patients showed a decrease in post-treatment urine cfDNA. In sum, more data will be necessary to better understand variations in urine cfDNA and to determine the utility of this biofluid in OC. Overall, further studies, including more patients and more timepoints, will clarify if there are significant differences between cfDNA levels in patients and controls in both biofluids, and allow to comprehend the patterns that cfDNA levels follow after treatment, verifying if these relate to treatment efficacy and/or clinical outcomes, such as recurrence development.

**Survival analysis and molecular discrimination of cholangiocarcinoma anatomical subtypes**

Inês Tavares^1^, Ilda P. Ribeiro^1,2,3^, Ricardo Martins^2,3,5^, Luísa Esteves^1^, Margarida Abrantes^2,3,4^, Maria Filomena Botelho^2,3,4^, José G Tralhão^2,3,5^, Joana B Melo^1,2,3^, Isabel M Carreira^1,2,3^

^*1*^*Cytogenetics and Genomics Laboratory, Institute of Cellular and Molecular Biology, Faculty of Medicine, University of Coimbra, Portugal;*^*2*^*iCBR-Coimbra Institute for Clinical and Biomedical Research – Area of Environment, Genetics and Oncobiology (CIMAGO), Faculdade de Medicina da Universidade de Coimbra, Portugal and CIBB - Center for Innovative Biomedicine and Biotechnology, University of Coimbra, Portugal,*^*3*^*CACC-Clinical Academic Center of Coimbra, Portugal.*^*4*^*Biophysics Institute, Faculty of Medicine, University of Coimbra, Portugal;*^*5*^*Surgery A, Surgery Department of Coimbra University Hospital, Faculty of Medicine, University of Coimbra, Coimbra, Portugal*

**Introduction:** Cholangiocarcinoma (CC) is a rare malignant tumor arising from the biliary tract epithelium. It is classified according to anatomical location as intrahepatic (ICC) and extrahepatic (ECC). These two types of CC seem to be biologically distinct as they have different risk factors, genetic alterations and clinical outcomes. The aim of this study was to differentiate between these two subtypes regarding genomic alterations and comparing the survival rates.

**Methods:** The minimum common regions of alteration were determined using the array-CGH results from 10 ECC and 13 ICC patients. The most important regions for the distinction were selected by a Variable Importance Plot in a bootstrapping scheme applied to a balanced set regarding the number of cases in each class. A two-class support vector machine (SVM) algorithm for statistical classification was applied to these data to test the ability of the selected regions to distinguish between the two classes. Survival curves were calculated using Kaplan-Meier method, comparing survival of ICC and ECC patients, and taking into account the alterations present in the regions used in the genomic model.

**Results:** Gain of 2q37.3 and Xp and loss of 3p, 11q11, 14q, 16q, Yp and Yq were the most common alterations observed ICC patients. Regarding the ECC patients, gain of 2q37.3 and 16p25.3 and loss of 3q26.1, 6p25.3–22.3, 12p13.31, 17p, 18q and Yp were the most common alterations shown within this group. The developed genomic model identified chromosomal regions that seem to enabled the distinction between ICC and ECC, namely 3q26.1, 6p25.3, 14q32.33 and Xq26, with an accuracy of 71.43%, 95% CI [43, 100]%. Regarding the survival rate, ECC patients survived, on average, 8 months less than ICC patients. Survival analysis related to the alterations in the region 6p25.3 revealed some differences consistent with the different survival rates of ICC and ECC patients.

**Conclusion:** Our findings support the idea that ICC and ECC may be two closely related but different biologic entities, and this model may be helpful for the development of novel therapeutic targets, taking a step towards a clinical personalized medicine.

This work was supported by CIMAGO and NRC-LPCC/CIMAGO grant

**Detection of copy number variations in the genome of fetus with ultrasound abnormalities**

Mariana Val^1^, Susana I. Ferreira^1^, Luís M. Pires^1^, Nuno Lavoura^1^, Joana Barbosa de Melo^1,2,3^, Isabel Carreira^1,2,3^

^*1*^*Laboratório de Citogenética e Genómica, Faculdade de Medicina da Universidade Coimbra, Coimbra, Portugal,*^*2*^* iCBR-Coimbra Institute for Clinical and Biomedical Research – Area of Environment, Genetics and Oncobiology (CIMAGO), Faculdade de Medicina da Universidade de Coimbra, Portugal and CIBB - Center for Innovative Biomedicine and Biotechnology, University of Coimbra, Portugal.*^*3*^*CACC-Clinical Academic Center of Coimbra, Portugal.*

**Context:** Fetal structural malformations and increased nuchal translucency (NT) can be related with genome alterations and have poor prognosis. Quantitative fluorescent polymerase chain reaction (QF-PCR) is routinely used to detect common aneuploidy in prenatal diagnosis (PND). Array comparative genomic hybridization (array-CGH) is a useful approach to detect submicroscopic imbalances and is recommended as the first tier genetic test when fetal ultrasound abnormalities are detected.

**Methods:** The present study includes 571 prenatal samples referred for invasive PND due to fetal structural malformations and/or increased NT. Rapid aneuploidy detection (RAD) was performed by QF-PCR and genomic imbalances were studied using Agilent 4x180K array-CGH platform.

**Results:** A positive result to RAD was obtained in 14% of the cases. Among the samples analyzed by array-CGH, 9% of the cases revealed pathogenic and/or likely pathogenic (P/LP) copy number variations (CNVs) and in 8.1% of the cases at least one variant of uncertain significance (VOUS) was detected. Regarding the VOUS detected, 85% were inherited, 7.5% were of unknown origin, 5% were *de novo* and in 2.5% of the cases an inherited and a *de novo* VOUS were observed. The most common phenotypes presented by fetus among P/LP CNVs were malformations of the nervous system (29%) with or without additional anomalies (38.5% and 61.5%, respectively) and malformations of the cardiovascular system (20.5%), of which 56% had additional anomalies. Regarding samples with VOUS, malformations of the nervous system and cardiovascular system were present in 22% and 12.5% of the cases, respectively. Isolated increased NT was observed in 6.8% of the fetus with P/LP CNVs and in 7.5% with VOUS.

**Conclusions:** In this study, QF-PCR revealed a positive result in 14% of the cases and array-CGH detected clinical relevant CNVs in 9% of the cases. Array-CGH allows detecting imbalances that would not be found using conventional cytogenetic technics, more quickly, increasing the diagnostic yield and decreasing the turnaround time. These results show the fundamental role of array-CGH in addition to QF-PCR in the genome study of fetus with ultrasound abnormalities.

**Errors of cell division causing an unusual genotype: three derivative chromosomes resulting from a paternal reciprocal translocation**

Marta Pinto^1^, Alexandra Mascarenhas^1^, Ana Jardim^1^, Cláudia Pais^1^, Joana B. Melo^1,2,3^, Isabel M. Carreira^1,2,3^

^*1*^*Laboratório de Citogenética e Genómica, Faculdade de Medicina da Universidade Coimbra, Coimbra, Portugal;*^*2*^* iCBR-Coimbra Institute for Clinical and Biomedical Research – Area of Environment, Genetics and Oncobiology (CIMAGO), Faculdade de Medicina da Universidade de Coimbra, Portugal and CIBB - Center for Innovative Biomedicine and Biotechnology, University of Coimbra, Portugal,*^*3*^*CACC-Clinical Academic Center of Coimbra, Portugal.*

**Introduction:** Reciprocal translocations are common events. Usually, when a supernumerary derivative chromosome is present a 3:1 segregation had occurred, being the partial aneuploidy associated to the chromosomal content of the derivative.

**Clinical Report:** We present a prenatal case, referred after the first trimester combined screening test. Conventional karyotyping revealed a t(17;22)(q24;q11.2), inherited from the father, and an extra supernumerary chromosome, identical to the der(22). To clarify the rearrangement oligoarray-CGH was carried out showing a duplication of the terminal long arm of chromosome 17(q24.3q25.3) of about 12.87 Mb and a duplication of part of the long arm of chromosome 22(q11.1q11.21) of about 2.88 Mb, confirming that the extra chromosome is, as predicted, a second der(22). The couple decided to terminate the pregnancy and refused further studies.

**Discussion/ Conclusion:** None of the possible segregation mechanisms occurring at Meiosis I explains the presence of an extra der(22). Therefore a secondary event is necessary to explain the unbalanced karyotype observed.

One possible mechanism is the occurrence of an alternate segregation of a paternal translocation in Meiosis I, followed by a nondisjunction in the second meiotic division. Another possibility is a postzygotic event, either a mitotic nondisjunction or an endoreplication, in a t(17;22)(q24;q11.2) balanced zygote.

Mitotic errors, endoreplication and anaphase lagging are relatively common during the initial postzygotic cell division but they are usually related to mosaicism, which was not observed in our case. Although a mosaic cannot be completely excluded, a non-disjunction at Meiosis II, in spite of it being a rare event, is the most probable mechanism to explain the extra der(22) in all the metaphases observed.

Errors during cell division and chromosome segregation generate genetic variability, with changes in chromosome content and, consequently, changes in the relative gene dosage. They are the primary cause of birth defects and infertility and also have important implications in oncogenesis.

**Paternal pericentric inversion of chromosome 4 and three recombinants in the offspring - Structural Variation of Human Genome**

Alexandra Mascarenhas^1^, Luís Miguel Pires^1^, Fabiana Ramos^2^, Marta Pinto^1^, Patrícia Paiva^1^, Joana Barbosa de Melo^1,3,4^, Isabel Marques Carreira^1,3,4^

^*1*^*Laboratório de Citogenética e Genómica – Faculdade de Medicina da Universidade de Coimbra, Portugal;*^*2*^*Serviço de Genética Médica, Hospital Pediátrico, Centro Hospitalar e Universitário de Coimbra;*^*3*^* iCBR-Coimbra Institute for Clinical and Biomedical Research – Area of Environment, Genetics and Oncobiology (CIMAGO), Faculdade de Medicina da Universidade de Coimbra, Portugal and CIBB - Center for Innovative Biomedicine and Biotechnology, University of Coimbra, Portugal,*^*4*^*CACC-Clinical Academic Center of Coimbra, Portugal.*

**Introduction:** Inversions are intrachromosomal structural rearrangements that result of a two-break event in the same chromosome and reinsertion of the chromosomal segment after a rotation of 180°. Pericentric inversions include the centromere and gametogenesis in chromosomal pericentric inversion carriers may give rise to the formation of a recombinant (rec) chromosomes. Inversions are related with phenotypic differences and adaptation in multiple species and they play a role as disease-causing mutations since they directly affect gene structure and because they predispose to other rearrangements in the offspring of inversion carriers.

**Case Report:** We describe a pericentric inversion of chromosome 4: inv(4)(p15.33q31.3) and three adverse fetal outcomes. This rearrangement was ascertained after a prenatal diagnosis because of ultrasound abnormalities. Conventional cytogenetics revealed a recombinant chromosome 4, derived from a paternal pericentric inversion, leading to partial trisomy 4p: 4(pter→p15.33) and partial monosomy of 4q: 4(q31.3→qter). Array-CGH analysis and chromosome analysis were performed in the following pregnancies. One of the fetus showed the presence of a different recombinant, with a deletion on the short arm of chromosome 4(pter→p15.33) and a duplication on the long arm of chromosome 4 (q31.3→qter). In the last gestation, array-CGH and chromosome analysis, performed in CVS sample, revealed the same imbalance found in the second fetus analysed [rec dup(4p)].

**Conclusion:** A careful genetic counselling before and after the genetic test, is very important to this couple, being really relevant that genetic counselling and genetic testing go together. The high frequency of recombinants in this family highlights the functional impact of inversions in the human genome. Our case report illustrates also the potential of inversions to have phenotypic consequences in humans and emphasize the importance of their inclusion in human genome variation studies, especially in carcinogenesis since sporadic *de novo* inversions are also known to have genetic risk factors for cancer susceptibility.

**Clinicopathological correlations with genomic and epigenetic signatures in oral cancer patients – preliminary data**

Ilda P. Ribeiro∗^1,2,3^; Ivana Martins∗^1,4^; Leonor Barroso^5^, Inês Tavares^1^; Francisco Marques^5^; Joana B. Melo^1,2,3^; Isabel M. Carreira^1,2,3^

∗both authors contributed equally to this work

^*1*^*Cytogenetics and Genomics Laboratory, Faculty of Medicine, University of Coimbra, Coimbra, Portugal;*^*2*^*iCBR-Coimbra Institute for Clinical and Biomedical Research – Area of Environment, Genetics and Oncobiology (CIMAGO), Faculdade de Medicina da Universidade de Coimbra, Portugal and CIBB - Center for Innovative Biomedicine and Biotechnology, University of Coimbra, Portugal,*^*3*^*CACC-Clinical Academic Center of Coimbra, Portugal,*^*4*^*Faculty of Sciences and Technology, University of Coimbra, Coimbra, Portugal;*^*5*^*Maxillofacial Surgery Department, Coimbra Hospital and University Centre, CHUC, EPE, Coimbra, Portugal;*^*5*^*Department of Dentistry, Faculty of Medicine, University of Coimbra, Coimbra, Portugal*

**Aims:** Oral cancer (OC) is characterized by the accumulation of genomic and epigenetic alterations, however, the identification of biomarkers with clinical impact remains a challenge. We aimed to establish the profiles of oral tumors, identifying concomitantly chromosomal regions and genes that frequently show copy number (CN) gains or losses, or hypermethylation, and to correlate this molecular data with patients’ clinicopathological features as well as with pre- and post-treatment plasma cell-free DNA (cfDNA) levels.

**Methods:** This study was conducted in 8 newly diagnosed oral tumor tissues and matched macroscopically tumor-free tissues from resection margins. Gingival tissues from healthy donors subjected to wisdom teeth removal were used as controls. Methylation-Specific Multiplex Ligation-dependent Probe Amplification (MS-MLPA) was performed using tumoral and non-tumoral tissues’ DNA for methylation profiling and CN variation (CNV) detection. Array Comparative Genomic Hybridization (aCGH) was also performed in tumoral DNA to assess CNV.

**Results and Conclusions**: We observed more CN losses than CN gains. The chromosomes most frequently displaying CN losses in aCGH were 1p, 10q, 11q, 12q, 15q, 16q, 17p, 17q, 18q, 19p, 19q, 20q, 21q, 22q, Yp, and Yq. Particularly, the most commonly deleted regions were 1p36.31-p32.3, 10q21.3-q22.2, 10q23.2-q25.1, 11q12.2-q13.2, 16q21-q23.1, 19q13.12-q13.43, 20q11.21-q13.2, 22q11.1-q12.3 and Yp11.32-p11.31. None of the detected CN gains were seen in more than 2 patients. Most CN losses in MS-MLPA were seen in the genes *CDKN2A* (9p21) in tumor tissue and *VHL* (3p25) in tumoral and non-tumoral tissue. The most hypermethylated genes in tumor tissue were *RARB* (3p24) and *WT1* (11p13). In sum, we identified commonly altered chromosomal regions, CNVs and hypermethylation in OC. The presence of alterations in apparently tumor-free tissues may indicate greater risk of relapse and a need for a more rigorous follow-up. The correlation between these novel molecular findings and clinicopathological data is pivotal to identify and validate precise and accurate biomarkers of prognosis and therapy response. A larger follow-up of these patients is necessary to correlate clinical and molecular data with cfDNA values.

**Correlation of signaling pathways and occurrence of metastases - a study in a cohort of Head and Neck Carcinoma patients**

Luísa Esteves^1^, Ilda Patrícia Ribeiro^1,2,3^, Leonor Barroso^4^, Francisco Marques^2,5,6^, Isabel Marques Carreira^3^, Joana Barbosa de Melo^3^

^*1*^*Cytogenetics and Genomics Laboratory, Faculty of Medicine, University of Coimbra, 3000–354 Coimbra, Portugal;*^*2*^*iCBR-Coimbra Institute for Clinical and Biomedical Research – Area of Environment, Genetics and Oncobiology (CIMAGO), Faculdade de Medicina da Universidade de Coimbra, Portugal and CIBB - Center for Innovative Biomedicine and Biotechnology, University of Coimbra, Portugal;*^*3*^*CACC-Clinical Academic Center of Coimbra, Portugal,*^*4*^*Maxillofacial Surgery Department, Coimbra Hospital and University Centre, CHUC, EPE, 3000–075 Coimbra, Portugal;*^*5*^*Department of Dentistry, Faculty of Medicine, University of Coimbra, 3000–075 Coimbra, Portugal;*^*6*^*Stomatology Unit, Coimbra Hospital and University Centre, CHUC, EPE, 3000–075 Coimbra, Portugal.*

**Context:** Head and neck squamous cell carcinoma (HNSCC) results from multiple alterations that involve several chromosomic regions, genes and cell signaling pathways. Understanding the molecular mechanisms of development and progression of these carcinomas is crucial for their diagnosis and prognosis as well as evaluating the risk of relapses and metastasis.

**Methods:** DNA from 62 tissue samples from HNSCC tumors was extracted and array Comparative Genomic Hybridization (aCGH) was performed. Chromosomic regions with copy number alterations in over 40% of patients were kept and the genes contained in those regions were then determined, resulting in a set of 24 genes. The overrepresented cell signaling pathways in our cohort (p < 0.01) were found using R3.5.1, for the patients that had developed metastasis and for those that had not, separately.

**Results:** Four signaling pathway were found to be overrepresented for the patients that developed metastasis and eight were found in the group of patients that did not. Only two of those were common to both groups (Melanoma and NOD-like receptor signaling pathway). However, even within the same pathway, the genes involved were not always the same. For the NOD-like receptor signaling pathway, *FADD, DEFB4B* and *DEFB4A* were present in both groups whereas *IKBKB* and *VDAC3* were only significantly altered in the patients without metastasis or relapse and *NAIP*, *GSDMD* and *SHARPIN* in the patients that developed them. For the Melanoma pathway *FGF3, FGF4, CCND1* and *FGF19* were shared, these genes co-localize to 11q13 which is a well described region associated with HNSCC. For the remaining eight signaling pathways, which were different between groups, 9 genes were unique to the pathways overrepresented in the patients with metastasis and 8 genes were only significantly altered in the patients without metastasis.

**Conclusion:** The two groups of tumors analyzed seem to be different at the molecular and the gene interaction levels, based on the most significant pathways for each, and some genes appear to be differentially altered. It is vital to understand the mechanisms behind metastization to improve the clinical outcome of the patients.

**Multiple basal cell carcinomas of the scalp after radiotherapy: genomic study in a case with latency period over 80 years.**

José C. Cardoso^1,2∗^, Ilda P. Ribeiro^2,3,4∗^, Francisco Caramelo^4,5^, Oscar Tellechea^1^, Joana B. Melo^2,3,4^, Isabel M. Carreira^2,3,4^

^∗^Both authors contributed equally to this work.

^*1*^*Dermatology Department, Coimbra Hospital and University Centre, Coimbra, Portugal,*^*2*^*CACC-Clinical Academic Center of Coimbra, Portugal;*^*3*^*Cytogenetics and Genomics Laboratory, Faculty of Medicine, University of Coimbra, Coimbra, Portugal;*^*4*^*iCBR-Coimbra Institute for Clinical and Biomedical Research – Area of Environment, Genetics and Oncobiology (CIMAGO), Faculdade de Medicina da Universidade de Coimbra, Portugal and CIBB - Center for Innovative Biomedicine and Biotechnology, University of Coimbra, Portugal;*^*5*^*Laboratory of Biostatistics and Medical Informatics, IBILI - Faculty of Medicine, University of Coimbra, Coimbra, Portugal*

Basal cell carcinoma (BCC), the most common form of skin cancer, has been linked mostly to ultraviolet radiation exposure, but ionizing radiation has also been implicated in the genesis of a subset of scalp BCCs in patients treated with radiotherapy for tinea capitis in childhood. In the latter, tumours are often multiple and occur after a latency period that may span several decades. We describe a case of multiple scalp BCCs after radiotherapy for tinea capitis arising after a latency period of more than 80 years, the longest reported to date.

A 93 year-old female patient presented with 4 BCCs, the largest one in the right temporal region (A) and the remaining in the right parietal region (B), vertex (C) and occipital area (D). Histological examination showed that lesion A was the more complex, corresponding to a BCC of mixed type, predominantly nodular and infiltrative, and focally micronodular. Lesions B and D were both nodular, and lesion C was a superficial BCC.

Genomic study with array comparative genomic hybridization revealed that lesion A had more imbalances than the remaining ones, with a predominance of copy number losses over gains. In spite of these differences, there were aberrations shared by all tumour samples, suggesting a common genetic signature (namely gains at 2q13, 5q13.2 and 14q21.2, and losses at 5p15.33, 8p21.3, 1q21.2, 2q44 and 7p22.3). Similarly, specific genomic signatures were shared between three and two of each of the four analyzed samples, such as tumors A and D that shared genomic aberrations related to apoptosis, namely gain at *PAK2* gene (3q29).

On the other hand, some lesions showed specific imbalances. For example, only tumor A presented loss at 2p22.3, where is mapped the *BIRC6* gene associated to regulation of apoptosis, and loss at 16q24.3, where is mapped *FANCA* gene, responsible for DNA repair and maintenance of chromosome stability.

Our findings suggest genomic imbalances common to different types of BCCs but also differences that could explain their heterogeneity in terms of histological subtype and biological potential and/or be the consequence of different times in the evolution of the lesions at the moment of sampling.

**Validation of a genomic approach to evaluate copy number alterations in Head and Neck Cancer**

Ana L. Santos^1^, Ilda P Ribeiro^1,2,3^, Luísa Esteves^1^, Francisco Marques^4,^ Leonor Barroso^5,^ Maria J Julião^6^, Isabel P Baptista^4^, Isabel M Carreira ^1,2,3^ and Joana B Melo^1,2,3^

^*1*^*Cytogenetics and Genomics Laboratory, Faculty of Medicine, University of Coimbra, Coimbra, Portugal,*^*2*^* iCBR-Coimbra Institute for Clinical and Biomedical Research – Area of Environment, Genetics and Oncobiology (CIMAGO), Faculdade de Medicina da Universidade de Coimbra, Portugal and CIBB - Center for Innovative Biomedicine and Biotechnology, University of Coimbra, Portugal;*^*3*^*CACC-Clinical Academic Center of Coimbra, Portugal,*^*4*^*Department of Dentistry, Faculty of Medicine, University of Coimbra, Coimbra, Portugal;*^*5*^*Maxillofacial Surgery Department, Coimbra Hospital and University Centre, CHUC, EPE, Coimbra, Portugal,*^*6*^*Department of Pathology, Coimbra Hospital and University Centre, CHUC, EPE, Coimbra, Portugal*

**Context:** Head and neck squamous cell carcinoma (HNSCC) encompasses oral cavity neoplasms whose main subtype is oral squamous cell carcinoma (OSCC) representing 90% of all oral cavity malignancies. The carcinogenic processes of this cancer leads to the accumulation of genetic imbalances.

We aim to validate a molecular approach to detect genomic imbalances in a 62 OSCC patients’ cohort.

**Methods:** We performed copy number alterations (CNA) analysis by two techniques, MLPA (MRC-Holland), using a HNSCC specific probe panel, and aCGH 180K (Agilent Technologies). Both techniques were used in the same cohort in order to obtain a more in-depth CNA analysis.

**Results:** In aCGH, the most frequent abnormalities were seen in chromosomes 8, 6p, and 11q. MLPA most common imbalances were seen in chromosomes 3, 8 and 11q, which presented high number of gains in *FADD*, *FGF4*, *CCND1* and *CTTN* genes. Common alterations detected in high frequency were observed in 3q29, 8q24.3 and 11q13 regions.

We validated the MLPA alterations shown in our analysis by aCGH data by frequency of alteration and by Weighted Kappa that showed higher agreement values for 11q13.3 region. Also, we found with aCGH alterations in high frequency with no MLPA coverage. These new regions of interest harbor 27 genes, namely *RNF168*, *FBXO45* and *APOD* related to tumorigenesis and metastasis processes.

**Conclusion:** The MLPA HNSCC specific panel is very useful for the detection of the most common CNA observed in our cohort, namely the ones in 11q13.3 region. Nevertheless, taking into account the results obtain by aCGH, other regions should be included in this panel to improve is usefulness.

**Inflammatory pseudotumor of the liver: a diagnostic challenge and case report**

Mónica Rodrigues^1^, Kayla Pereira^1^, Ana Rita Neto^1^, Regina Leite^1^, João Pedro Barros^1^

^*1*^*Radiotherapy Department of Portuguese Institute of Oncology of Coimbra*

The Inflammatory Pseudotumor of the Liver (IPTL) is an uncommon benign tumor in occidental countries, appearing more often in men than in women. The lesion may be as large as 25 cm in the major axis. The etiology and pathogenesis of IPTL remains unknown. However, it has been recently suggested, that a IgG4-related LIP might be a possible cause.

This tumor constitutes an important diagnostic challenge as it presents in a sub-acute manner, mimicking malignant conditions, without specific analytical or imaging criteria. Differentiating between IPTs and other focal hepatic lesions remains a major problem. The most common symptoms of IPTL are abdominal pain, fever and weight loss.

In general, imaging study fails to distinguish this lesion from malignant tumors. Ultrasound and CT scans are not specific, often with variable patterns of echogenicity of a liver mass that resembles hepatocellular cancer or an hepatic abscess.

If an atypical solid mass is found in the liver, this diagnosis should be considered as a potential diagnosis.

The histological examination is the gold-standard for the diagnosis of Inflammatory Pseudotumor of the Liver. If a liver biopsy is performed, it is possible to avoid unnecessary hepatectomies and increased morbidity.

In the majority of cases, the prognosis is excellent with complete resolution of the lesion without hepatic resection.

We present a 53-year old male with asthenia and abdominal pain in 2013. Imaging studies revealed an intra-hepatic mass. Liver biopsy was performed, revealing IPTL. No treatment was conducted. The hepatic lesion has since been controlled by MRI, with progressive reduction on size. The patient remains disease free to the present day.

**Radiotherapy in the treatment of testicular seminomas: a single institutional experience**

Luísa Rolim^1^, Bruno Fernandes^1^, Carolina Carvalho^1^, António Silva^1^, Ana Cleto^1^, Margarida Borrego^1^

^*1*^*Department of Radioteraphy, Coimbra Hospital and University Center, Portugal*

**Objectives:** Germ-cell testicular tumors correspond to less than 1% of male malignancies, 60% of which are seminomas. Within this group, about 80% of patients are in stage I. These tumors have high radiosensitivity, presenting a cure rate of 90% and probability of recurrence, after 5 years, less than 0.3%. Treatment in pure seminomas, stages I_A_ and I_B_, comprises radical orchidectomy, followed by active surveillance or adjuvant treatment, including chemotherapy (1 - 2 cycles of carboplatin) or radiotherapy (RT). This study aimed to evaluate patients with classic testicular seminoma in stage I undergoing radical orchidectomy and postoperative RT.

**Methods:** This is a retrospective study that analyses 20 patients treated between 2002 and 2011, with radical orchidectomy followed by RT. RT was administered with 25Gy/20F (iliac, internal and external, ipsilateral, and paraaortic lymph nodes) by 2010, and from this date, with 20Gy/10F (paraaortic lymph nodes). Toxicity was evaluated by CTCAE (v5.0). The curves of overall survival (OS) and disease-free survival (DFS) were calculated according to the Kaplan-Meier method.

**Results:** The study included 20 patients aged between 26 and 39 years (median 33 years); 55% were classified at stage I_A_ and 45% in stage I_B_. Preoperative serological markers were obtained in 16 patients, with only 3 presenting an increase in LDH (12,5%). All patients completed treatment. During RT, the most frequent toxicity was gastrointestinal G_1–2_ (75%), followed by radiodermatitis G_1–2_ (15%); only 4 patients had urological symptoms. There was no acute toxicity of G_3–4_ or chronic toxicity. Follow-up time ranged from 59 to 195 months (median 114.5 months), with OS of 100% at 3, 5 and 10 years, and DFS of 100% at 3 years and 95% at 5 and 10 years. Only one patient had a local recurrence.

**Conclusions:** Treatment was, overall, well tolerated, in the short and long-term. The excellent survival rate is in accordance with that described in the literature, corroborating the use of adjuvant RT in stages I_A_ and I_B_. These results reveal the benefit of RT in the local control of these tumors and indicate its importance as a complementary first-line treatment.

**Influence of oral breathing on the occlusal patterns**

Luísa Maló^1^, Ana R. Silva^2^, João C. Ribeiro^3^, José P. Martinho^4^

^*1*^*Institute of Orthodontics, Dentistry Department, Faculty of Medicine, University of Coimbra, Portugal;*^*2*^*Dentistry Department, Faculty of Medicine, University of Coimbra, Portugal,*^*3*^*ORL, University Hospital Center of Coimbra, Portugal; Faculty of Medicine, University of Coimbra, Portugal; iCBR, Coimbra, Portugal,*^*4*^*Institute of Endodontics, Dentistry Department, Faculty of Medicine, University of Coimbra, Portugal.*

**Aims/Context** – Nasal obstruction is quite significant in the Portuguese population, having a high prevalence in the pediatric population. Children with oral breathing do not sleep well due to blocked airways, which can negatively affect their growth and academic performance. Oral breathing is considered a risk factor for malocclusion, as it alters the physiological balance of growth, leading to morphological changes in dental and craniofacial parameters. Therefore, the aim of this study was to evaluate the relationship between respiratory function and occlusal patterns present in a pediatric population.

**Methods** - A total of 45 children attending Orthodontic consultations were evaluated regarding respiratory function using the PNIF apparatus. They were also evaluated regarding systemic diseases; craniofacial, dental and functional patterns using the ROMA index.

**Results** – The study determined that all the tested children had poor respiratory function, with PNIF values lower than those proposed for each age group. An association between oral breathing and class II of Angle, middle palate, open bite, and maxillary hyperdevelopment or mandibular hypodevelopment was verified. No statistically significant difference was found between oral breathing and dental crowding, maxillary hypodevelopment or mandibular hyperdevelopment, anterior and posterior crossbite, and vertical overbite greater than 5.

**Conclusions** – All the studied indviduals, although with mostly nasal respiration, had poor respiratory function, requiring further study of the etiological factors and a multidisciplinary treatment, involving namely orthodontics and otorhinolaryngology. The study confirmed the presence of oral breathing in individuals with a tendency for openbite with maxillary hyperdevelopment or mandibular hypodevelopment which leads to a class II of Angle occlusal relationship.

**Effect of orthodontic tooth movement on dental pulp mineralization process.**

José Pedro Martinho^1^, Eurico Serrano^2^, João Garcia^3^, João Brochado^3^, Luísa Maló^4^

^*1*^*Institute of Endodontics of Faculty of Medicine, University of Coimbra, Portugal;*^*2*^*Biochemistry Department, Faculty of Science and Technology, University of Coimbra, Portugal;*^*3*^* Dentistry Department, Faculty of Medicine, University of Coimbra, Portugal;*^*4*^* Institute of Orthodontics of Faculty of Medicine, University of Coimbra, Portugal.*

**Aims/Context:** Dental pulp stem cells in response to a variety of environmental signals are capable of generating new stem cells and engage in specific differentiation pathways, of which the odontoblastic is essential for the maintenance of pulp homeostasis. As a survival mechanism, the pulp-dentin system responds to an aggression not only with proliferation and differentiation of dental pulp stem cells but also with the production of mineralized tissue inside the dental pulp. The aim of this study was to determine the effects of orthodontic forces on the dental pulp mineralization process.

**Methods:** Pre-molars subjected to orthodontic forces were divided into 4 groups (control; and 1, 2 and 3 weeks of orthodontic movement) and subjected to histologic and immunohistochemistry analysis with specific mineralized tissue antibodies Osteopontin and Osteocalcin.

**Results:** At weeks 2 and 3 was observed the formation of mineralized tissue in the dental pulp; and an inflammatory response with differentiation of adipose tissue. The expression of Osteopontin was the same in all experimental groups, while was observed an increase in the expression of Osteocalcin at week 3.

**Conclusions:** In relation to Osteopontin, it can be infer that the dental pulp expresses this protein as an attempt to contradict the defense mechanism that is the pulp mineralization. The increased expression of osteocalcin in week 3 allow us to conclude that it is at this later period that the differentiated odontoblast-like cells exist in greater numbers and are metabolically active.

**Using less than 10% formalin fixed tissue samples in vimentin immunohistochemical detection: possible impact on oncobiological analysis?**

Raquel Graça-Lopes^1^, Mariana Almeida^1^, Manuela Novo^2^, João Palma^1,3^, Mário Matos^1^, Carina Ladeira^1^, Amadeu Borges-Ferro^1^

^*1*^*Escola Superior de Tecnologia da Saúde de Lisboa, Instituto Politécnico de Lisboa. Portugal;*^*2*^*Hospital Fernando Fonseca E.P.E., Amadora, Portugal;*^*3*^*Hospital de Vila Franca de Xira, Vila Franca de Xira, Portugal*

**Context:** There are very few circumstances where the diagnosis of malignancy is made in the absence of histopathological confirmation. For prognosis and therapeutic guidance, it is also essential to support all decisions with knowledge of the oncobiology provided by immunohistochemistry and other molecular methodologies. Regarding this goal, it is essential to obtain/preserve adequate diagnostic material. In case of tissue samples, the most widely used fixative is 10% neutral buffered formalin. However, this concentration is not supported by consistent scientific evidence.

**Aim:** To compare immunohistochemical results considering less than 10% formalin concentrations, in order to support the proper use of this reagent and also to explore the possibility of decreasing the formalin use, since its toxicity is known.

**Methods:** Three formalin concentrations (10; 7,5; 2,5%) and 70% ethanol (representing non-formalin fixation) were used to fix, for 48 hours at room temperature, similar sized human placenta samples, with a cold ischemia time of 15 minutes. For each fixative, 30 paraffin blocks were prepared, which, after sectioning, were subjected to vimentin (NCL-L-VIM-V9) immunohistochemical detection (Ventana Benchmark GX - OptiView DAB), has this structural protein is commonly used as a fixation quality biomarker. Immunohistochemical results were assessed by two experts using the Global Immunohistochemistry Score (GIS) providing a 0–100 points score. The GIS results were analysed using descriptive statistics and Kruskal-Wallis test with pairwise comparison.

**Results:** The results were (mean-standard deviation): 2,5% formalin (90,10–6,94); 7,5% formalin (86,77–7,22); 10% formalin (82,94–9,62); 70% ethanol (84,67–9,49). The Kruskal-Wallis test revealed statistical differences between 2,5% formalin and all the other samples (p < 0,05). The internal consistency between the two observers was considered satisfactory (α=0,72).

**Conclusions:** Results sustain that 2.5% formalin provided best immunohistochemical results in this context. Even considering the exploratory type of this research, it is possible to consider the reduction of formalin concentration used in fixation.

**Improving peripheral venous catheterization-related outcomes in oncology patients: an action research study in Portugal**

Paulo Santos-Costa^1,2^, Liliana B. Sousa^2^, Cristina Costeira^3^, Filipe Santos^3^, Anabela Salgueiro-Oliveira^2^, Pedro Parreira^2^, Margarida Vieira^4^, João Graveto^2^

Universidade Católica Portuguesa - Instituto de Ciências da Saúde, Porto, Portugal, ^2^Unidade de Investigação Ciências da Saúde: Enfermagem da Escola Superior de Enfermagem de Coimbra, Portugal;^3^Instituto Português de Oncologia de Coimbra Francisco Gentil, E.P.E., Aptd. 2005 Coimbra, Portugal;^4^Centro de Investigação Interdisciplinar em Saúde, Universidade Católica Portuguesa, 4169-00 Porto, Portugal

**Aims/context:** Peripheral intravenous catheterization (PIVC) is not a risk-free procedure with complications rates as high as 90%. In the literature, evidence on the incidence and impact of such procedure in oncology patients is scarce. Thus, we intend to identify current clinical practices and technologies in use during the PIVC of oncology patients. Based on the initial findings, a multimodal intervention will be implemented and its effectiveness in reducing associated complications will be assessed.

**Methods:** Through Action Research method, three major phases of activities were defined: i) Planning phase, where current PIVC-related practices, technologies in use and complication rates will be identified; ii) Action phase, with the implementation of a multimodal intervention to align PIVC-related practices to the latest scientific evidence; iii) Reflection phase, discussing the project's results with the ward team and the institution's board. This study will be conducted at the Instituto Português de Oncologia de Coimbra following approval from the Administration Board and Ethical Committee.

**Results:** An Observational Prospective study is being carried with the support of the general surgical department's medical consultant and nursing manager. Clinically stable patients age ≥18 years and with the indication for PIVC insertion for a period ≥24 hours are being recruited for study participation. Patients who received immunosuppressive or chemo/radiotherapy treatment in the three months prior to the study recruitment date are automatically excluded. Daily data collection is being undertaken focused on PIVC-related variables (e.g., gauge, selected site) and outcomes (e.g., phlebitis and infiltration rates), patient demographical and clinical variables.

**Conclusions:** We expect that the multimodal intervention to be developed substantiates higher quality and safety standards during PIVC-related care provision, with the potential to be replicated in other oncology settings.

**Pain Evaluation for Health Professionals - Impact on Patient Care**

Rui Garcia^1^, Cristina Santos^1^, Catarina Rodrigues^1^

^*1*^*Haematology Service- Centro Hospitalar e Universitário de Coimbra*

**Introduction and Goals:** Pain is a symptom that affects the quality of life of millions of people around the world, constituting the main reason for the demand for health care by the population

It is a morbidity and mortality factor associated with longer-lasting hospital stays and increased consumption of health resources.

In this study tha authors aim to:

1) Analyze the use of tools for pain assessment in a hospital environment

2) Document the main analgesic strategies used

3) Evaluate the quality of the approach of the hospitalized patient with pain by health professionals

**Methods:** A retrospective observational cross-sectional study (1 month) was designed. The study population included 2 groups

N1 = 125 health professionals from an hospital service

N2 = 192 patients hospitalized for at least 72 hours

Exclusion criteria considered: length of stay less than 72 h, complete absence of procedural records

Statistical analysis: calculation of frequencies and medians

**Results:** Less than half of the reviewed clinical files (47.4% medical records and 45.3% nursing records) document pain on admission and less than one-sixth of the clinical records (13.5%) report the type of analgesia actually administered and the therapeutic response obtained-

Only about half of the therapeutic tables (50.5%) include indications for assessing the patient's pain during hospitalization, although 11.5% have prescription painkillers

Most hospitalized patients experience pain during hospitalization and pain complaints have been poorly documented, conditioning their therapeutic approach

Best practices in addressing pain have been inconsistently applied

**Conclusion:** The proper assessment and the institution of individualized analgesia that contemplates the different aspects of pain should be considered as an integral part of daily clinical routines

An integrated approach to pain is essential for defining analgesic strategies, improving the quality of care provided to inpatients

Study shows the need to implement surveys and pain assessment scales for hospitalized patients in the clinical interview and adequately document complaints whenever pain is confirmed, it should be continuously monitored and treated.

**Multidisciplinary meeting to support family of oncological patients**

Cristina Santos^1^,Catarina Rodrigues^1^, Rui Garcia^1^

^*1*^*Serviço de Oncologia -Centro Hospitalar e Universitário de Coimbra*

**Introduction and Goals:** In holistic care for the severely ill person, we cannot fail to identify family problems and concerns that will be the key to adapt the person to the disease.

Most of caregivers in palliative care emerge from family. Their roles have an impact on the life and quality of care provided. Health teams must be aware of these changes and plan supportive interventions, with measures that prevent the collapse of his life.

**The aims are:**-Hold multidisciplinary meeting with professionals and family.

-Assess family needs, opportunities and resources to support safe home return.

-Promote symptomatic relief and fulfillment of the patient's wishes.

**Methodology:** An exploratory study was elaborated, with quantitative data. A multidisciplinary premeeting questionnaire was applied to all families of patients admitted to oncology service and at the end of meeting a new questionnaire was applied. Responses were voluntary, anonymous and confidential.

**Results:** Fifty-two pre and post meeting questionnaires were applied. Regarding pre meeting questions 86% of the families wanted information about the patient's clinical situation and how to take care of patient. In post meeting questions 100% of the families expressed satisfaction in holding the meeting, considering it a necessity and an asset and a perk.

This multidisciplinary meeting clarified, reassured and relieved family's anguish. At the end 90% of the families did not express doubts or difficulties regarding how to take care of their patient, allowing safe return home.

It was evidenced that they are clear about the patient's condition and how to intervene according to the needs of each moment. Families also highlight the fact that this is a meeting with a multidisciplinary team in which the family is considered an active member.

**Conclusion:** The families acquired knowledge and skills allowing them to make decisions about how to care the patient, with fewer referrals to palliative care facilities. The results confirm the need of family involvement in care process of their ill member, contributing to improvement of the quality of life of all ando f the care provided, namely with the satisfaction of the patient's wishes.

**The use of chlorhexidine gluconate dressings in central venous catheters**

Catarina Rodrigues^1^, Cristina Santos^1^, Micaela Rodrigues^1^

^*1*^*Haemathology Service- Centro Hospitalar e Universitário de Coimbra*

**Introduction:** The use of chlorhexidine gluconate dressings is recommended to protect the insertion site of short-term central venous catheters (CVC), and is specifically indicated to reduce infections associated with CVC. The dressing consists of transparent adhesive film and a gel pad impregnated with 2% chlorhexidine gluconate, an antiseptic agent with broad spectrum antimicrobial / antimycotic activity, and also contains a plastic fixing device, on a breathable base in adhesive. silicone, less aggressive to the skin, which when replacing the fixation points reduces the entrance doors to microorganisms.

**Methods:** This study aims to evaluate the effectiveness of using chlorhexidine gluconate dressings in CVC, as well as its importance in reducing CVC-associated infection in a clinical hematology service. This is a descriptive and quantitative study, with a non-probabilistic sample, with a number of 32 patients, between April and June of 2019. The questionnaires were completed by the nurses who performed the treatment at the CVC insertion site according to the training performed. All ethical procedures inherent to the study were followed.

**Results:** The results revealed that patients had a predominant pathology of multiple myeloma, with the subclavian vein being the preferred site for CVC insertion. The dressings applied in comparison with the semi-permeable transparent polyurethane films, placed previously, allowed to increase the replacement frequency to 7 days in half of the evaluations. In the rest, the dressing was replaced because it was damp, loose or dirty and inflammatory signs were observed in 31.8% of the evaluations compared to the previous ones, which was 50%.

**Conclusion:** The use of chlorhexidine gluconate dressings requires less manipulation, therefore less risk of infection. The gel allows a continuous application of chlorhexidine gluconate at the puncture point and as they are transparent dressings they allow visualization of the insertion site without removing it. The gel was able to absorb liquids (sweat, blood or exudate) without interfering with antiseptic activity. In turn, the fixation device allows strong adhesion and is less aggressive to the skin.

**ROS1 positive Non-Small Cell Lung Cancer clinical case**

Sónia Silva^1^, Salvato Feijó^1^

^*1*^*Centro Hospitalar de Leiria*

**Introduction:** The identification of molecular targets has become indispensable for the treatment of lung cancer and has revolutionized its prognosis. However, the percentage of mutated patients who can benefit from these therapies is low. ROS1 gene rearrangement is present in only 1–3% of the lung adenocarcinomas.

**Clinical case:** A 67 year old non-smoker woman presented to the emergency department with dyspnea and cough for the last 3 months. She had a chest CT scan with a 41x47 tumor lesion in the left lower lobe, homolateral pleural effusion and multiple adenopathies.

Bronchoscopy (27/07/2018) revealed tumor lesion with obliteration of the left lower lobe bronchus. Biopsies revealed lung adenocarcinoma.

On August 14, she was hospitalized for serious worsening of dyspnea. CT angiography showed a significant worsening of the left hilar mass with left principal bronchus obliteration with atelectasis, worsening of the pleural effusion and bilateral lung metastases. She repeated bronchofibroscopy (21/08) with significant worsening with infiltration of practically all left main bronchus and total obstruction of the lobar orifices.

Awaiting molecular study she started chemotherapy with cisplatin and pemetrexed on August 23 and was discharged with PS2 and on oxygen therapy. Revealing to be ROS1 positive she started Crizotinib on 28/09. After 2 weeks of treatment, there was significant radiological and symptomatic improvement (PS1 and no more need for oxygen therapy). Currently, more than one year after starting therapy, she has PS 0 and CT scan revealing complete response.

**Conclusion:** We describe a case of an agressive lung cancer with complete response maintained after 1 year of therapy.

